# Epstein-Barr virus suppresses N^6^-methyladenosine modification of TLR9 to promote immune evasion

**DOI:** 10.1016/j.jbc.2024.107226

**Published:** 2024-03-25

**Authors:** Xiaoyue Zhang, Zhengshuo Li, Qiu Peng, Can Liu, Yangge Wu, Yuqing Wen, Run Zheng, Chenxiao Xu, Junrui Tian, Xiang Zheng, Qun Yan, Jia Wang, Jian Ma

**Affiliations:** 1 Hunan Cancer Hospital and the Affiliated Cancer Hospital of Xiangya School of Medicine, Central South University, Changsha, Hunan, China; 2 Cancer Research Institute, School of Basic Medical Science, Central South University, Changsha, Hunan, China; 3 NHC Key Laboratory of Carcinogenesis, Key Laboratory of Carcinogenesis and Cancer Invasion of the Chinese Ministry of Education, Hunan Key Laboratory of Nonresolving Inﬂammation and Cancer, Hunan Key Laboratory of Cancer Metabolism, Changsha, Hunan, China; 4 Department of Pathology, Affiliated Hospital of Guilin Medical University, Guilin, Guangxi, China; 5 Department of Clinical Laboratory, Xiangya Hospital, Central South University, Changsha, China; 6 Department of Immunology, Changzhi Medical College, Changzhi, Shanxi, China

**Keywords:** Epstein-Barr virus, METTL3, TLR9, m^6^A modification, immune evasion

## Abstract

Epstein-Barr virus (EBV) is a human tumor virus associated with a variety of malignancies, including nasopharyngeal carcinoma, gastric cancers, and B-cell lymphomas. N^6^-methyladenosine (m^6^A) modifications modulate a wide range of cellular processes and participate in the regulation of virus-host cell interactions. Here, we discovered that EBV infection downregulates toll-like receptor 9 (TLR9) m^6^A modification levels and thus inhibits TLR9 expression. TLR9 has multiple m^6^A modification sites. Knockdown of METTL3, an m^6^A “writer”, decreases TLR9 protein expression by inhibiting its mRNA stability. Mechanistically, Epstein-Barr nuclear antigen 1 increases METTL3 protein degradation *via* K48-linked ubiquitin-proteasome pathway. Additionally, YTHDF1 was identified as an m^6^A “reader” of TLR9, enhancing TLR9 expression by promoting mRNA translation in an m^6^A -dependent manner, which suggests that EBV inhibits TLR9 translation by “hijacking” host m^6^A modification mechanism. Using the METTL3 inhibitor STM2457 inhibits TLR9-induced B cell proliferation and immunoglobulin secretion, and opposes TLR9-induced immune responses to assist tumor cell immune escape. In clinical lymphoma samples, the expression of METTL3, YTHDF1, and TLR9 was highly correlated with immune cells infiltration. This study reveals a novel mechanism that EBV represses the important innate immunity molecule TLR9 through modulating the host m^6^A modification system.

Epstein-Barr virus (EBV), a large double-stranded DNA virus belongs to gamma-herpes virus subfamily. It latently infects approximately 95% of the world’s adult population and is the first human virus identified to be associated with human cancers, including nasopharyngeal carcinoma, gastric cancer, and several types of lymphoma (1, 2). There are two stages of EBV infection: latent infection and lytic infection. EBV establishes latent infection in the human host by infecting B cells. Latent infections are categorized as latent 0, I, II, and III (3), and B lymphocytes are preferred target cells for EBV to establish latent infections (4). The ability of EBV to transform primary B cells into proliferating lymphoblastoid cells demonstrates the important role of EBV in B-cell malignancies. The three major B-cell malignancies associated with EBV are Burkitt's lymphoma, Hodgkin's lymphoma, and diffuse large B-cell lymphoma (3).

Epstein-Barr nuclear antigen 1 (EBNA1) is a sequence-specific DNA-binding protein encoded by EBV that is expressed in all EBV-associated tumors and viral latency (1). During the latent phase, EBNA1 tethers the viral genome to the host chromosome promoting replication and persistence of the EBV genome. EBNA2, EBNALP, 3A, 3B, and 3C act as transcription factors regulating host and viral genes expressions (5, 6). EBNA1 regulates viral and host genes transcription and interacts with host proteins involved in viral oncogenesis-associated diseases (7). EBNA1 transcriptionally activates Survivin, an apoptosis suppressor, to promote tumorigenesis (8). In addition, EBNA1 is involved in regulating the interaction with the ubiquitin-modifying enzymes STUB1 and USP7 through two SUMO-interacting motifs, which favors DNA binding and episome maintenance function (9).

N^6^-methyladenosine (m^6^A) modification is one of the most abundant RNA modifications on eukaryotic mRNAs (10, 11, 12). The m^6^A modification is a dynamically reversible process, which is controlled by the methyltransferase complex (METTL3, METTL14, and WTAP) and the demethyltransferase (FTO and ALKBH5). Furthermore, specific RNA-binding proteins recognizing m^6^A modification sites, such as YTHDF1, 2, 3 (13, 14), YTHDC1, 2 (15, 16, 17), eIF3 (18), IGF2BP1/2/3 (19) and heterogeneous nuclear ribonucleoprotein (hnRNPA2B1 and HNRNPC) (14, 20), can regulate a wide range of mRNA fates. m^6^A modification patterns are engaged in regulation of multiple RNA and DNA viral infection processes, such as SARS-CoV-2 (21), herpes virus type 1 (22), hepatitis B virus (HBV) (23), enterovirus 71 (24), influenza A virus (25). m^6^A modification has been reported to promote viral replication and decrease antiviral immune signaling (26, 27). m^6^A modifications also have opposing effects in viral replication, such that the m^6^A reader YTHDF1 inhibits EBV replication and promotes EBV RNA decay (28).

The mammals sense exogenous stimuli through pattern-recognition receptors. Among them, toll-like receptors (TLR) expressed predominantly on antigen-presenting cells, which trigger innate immune responses by recognizing pathogen-associated molecular patterns (29). TLR9, localized to intracellular membrane compartments, such as the endoplasmic reticulum, endosomes and lysosomes, recognizing unmethylated CpG-DNA of bacterial or virus DNA (30, 31), which recruits the adaptor MyD88 and induces a cascade of nuclear translocations by the transcription factor NF-κB (32). TLR9 recognizes a variety of DNA viruses and elicits antiviral responses, and the recognition of CpG-containing oligodeoxynucleotides (CpG-ODNs) induces type I interferon (IFN) production by plasmacytoid dendritic cells (pDCs) (33, 34, 35). EBV has developed strategies to evade immune detection and manipulate the immune system by altering multiple cellular functions. It has been shown that during latent phase, EBV LMP1 strongly inhibits *TLR9* mRNA and protein expression in primary human B cells, thereby suppressing TLR9 function in interleukin (IL)-6, tumor necrosis factor (TNF)-α and immunoglobulin G (IgG) generation (36). Moreover, EBV infection significantly inhibits the TLR9 induced B cell response (37). During the lytic phase, the EBV protein BGLF5 represses the expression of TLR9 through RNA degradation in human B cells (38). Suppression of the TLR9-induced host immune response is an important immune evasion strategy employed by EBV. In addition, synthesis of TLR9 agonists has been suggested as potential anticancer therapeutics. Clinical trials had shown that CpG ODNs are effective in the therapy of various cancers. Modified TLR9 agonists are able to induce and recruit antitumor T cells to reverse resistance to PD-1 blockade therapy in advanced melanoma patients by triggering strong IFN responses (39). To take it a step further, inhalation of TLR9 agonists in lung cancer patients leads to lung remodeling, which results in CD8^+^ T cell infiltration of the tumor, dendritic cell expansion, and antibody production (40).

Recently, m^6^A modifications have been found to affect EBV transcriptional reprogramming (41) or participate in the regulation of EBV replication (42), but how they regulate viral-host interactions and what role they play in this process remains unclear. In the current study, we revealed EBV infection decreases TLR9 m^6^A modification and its function, and the underlying mechanism is related to EBNA1 mediated METTL3 protein degradation. We also revealed the possible antitumor immunity functions of METTL3 inhibitor and TLR9 agonist, which could be a novel treatment strategy for the EBV-associated malignances.

## Results

### EBV infection suppresses *TLR9* m^6^A modification and expression levels

**Figure 1:**
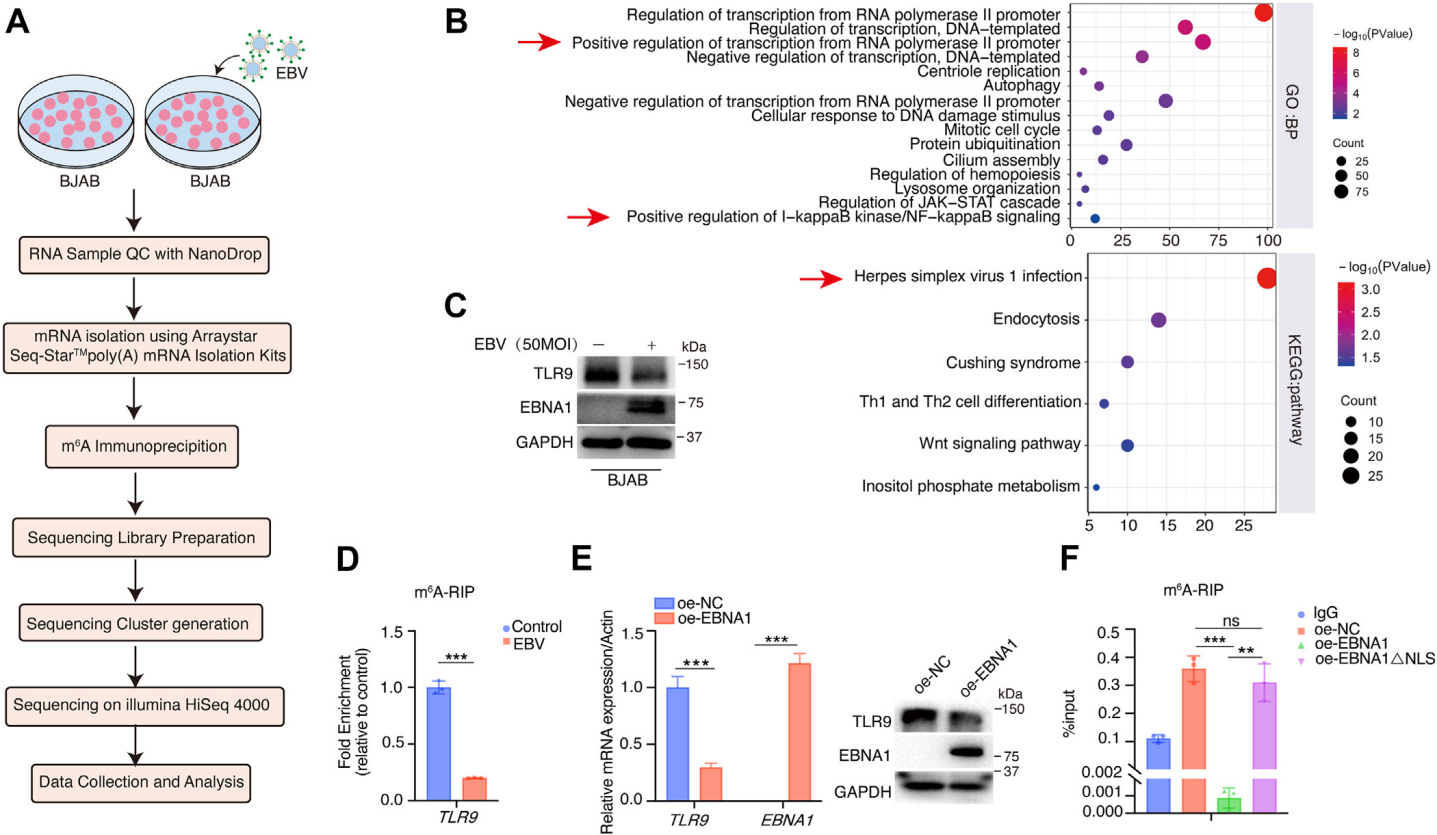
**EBV infection suppresses TLR9 m**^**6**^**A modification and expression.***A*, schematic diagram of the MeRIP-seq protocol used to identify RNA m^6^A profile changes in BJAB cells upon EBV infection. *B*, GO and KEGG pathway enrichment results for downregulated m^6^A-modified genes upon EBV infection (*p* ≤ 0.05, date from our previous study: #GSE133936). *C*, BJAB cells were infected with 50 MOI EBV for 48 h. TLR9, EBNA1, and GAPDH protein expressions were examined. *D*, m^6^A -RIP-qPCR analysis of *TLR9* mRNA expression in BJAB cells upon EBV infection. Control group was used for negative control. *E* and *F*, Flag-TLR9 was pretransfected into HEK293 cells for 6 h, then, Flag-NC, Flag-EBNA1, or Flag-EBNA1△NLS plasmids were respectively transfected for 24 h. The mRNA and protein expressions were measured by RT-qPCR and Western blotting (*E*). The m^6^A abundance of *TLR9* mRNA was analyzed by m^6^A -RIP-qPCR (F). Three independent experiments were performed, and data are shown as the mean ± SD. ∗∗*p* < 0.01, ∗∗∗*p* < 0.001, ns, not significant. EBNA1, Epstein-Barr nuclear antigen 1; EBV, Epstein-Barr virus; GO, Gene Ontology; KEGG, Kyoto Encyclopedia of Genes and Genomes; m^6^A, N^6^-methyladenosine; MeRIP, methylated RNA immunoprecipitation; MOI, multiplicity of infection; TLR, toll-like receptor; RIP, RNA immunoprecipitation.

To investigate the effect of EBV on m^6^A modification of the host cell transcriptome, we used EBV to infect BJAB cells. RNAs were collected after EBV infection for methylated RNA immunoprecipitation (MeRIP)-seq to assay the RNA m^6^A profiles change (Fig. 1*A*). The raw sequencing data of the MeRIP-seq had been deposited in National Center for Biotechnology Information Gene Expression Omnibus (NCBI GEO) under accession number GSE133936. We analyzed the sequencing data and found that EBV infection has a significant impact on the RNA m^6^A profiles of host cells (43). Gene Ontology analysis showed that the host genes whose m^6^A levels are repressed by EBV infection were mainly involved in the transcriptional regulation of RNA polymerase Ⅱ, the transcriptional regulation of DNA template, the replication of centriole, and autophagy process, and so on. Kyoto Encyclopedia of Genes and Genomes pathway analysis suggested that these host genes are involved in herpes simplex virus 1 infection, endocytosis and other processes (Fig. 1*B*). Among these genes, TLR9, a pattern-recognition receptor involved in innate immune response, attracted our attention. Fig. S1, *A* and *B* indicates a successful EBV infection in BJAB cells. EBV infection suppressed TLR9 protein expressions (Fig. 1*C*) as well as its RNA m^6^A levels (Fig. 1*D*). We next examined the TLR family genes *TLR1* to *TLR10* mRNA expression levels. Results from reverse transcription-quantitative PCR (RT-qPCR) and Western blotting analysis revealed that the mRNA expression levels of *TLR1* to *TLR10* and the protein expression levels of TLR3, TLR7, and TLR8 were not affected by EBV infection (Fig. S1, *C* and *D*). EBNA1 is essential for the maintenance of EBV latent infection, and also interacts with several cellular proteins to regulate multiple genes transcription (44, 45, 46). We found that EBNA1 could significantly inhibit TLR9 expression levels (Fig. 1*E*). We constructed a truncated variant of EBNA1 lacking the nuclear localization signaling domain (Fig. S1*E*). m^6^A-RNA immunoprecipitation (RIP) analysis revealed that nuclear localization signaling-deficient EBNA1 fails to suppress TLR9 m^6^A modification levels (Fig. 1*F*) and TLR9 protein expression (Fig. S1*F*). These results suggest that EBV suppresses TLR9 m^6^A modification and expression levels possibly through EBNA1, at least partly.

### Knockdown of METTL3 inhibits TLR9 and downstream molecules

**Figure 2:**
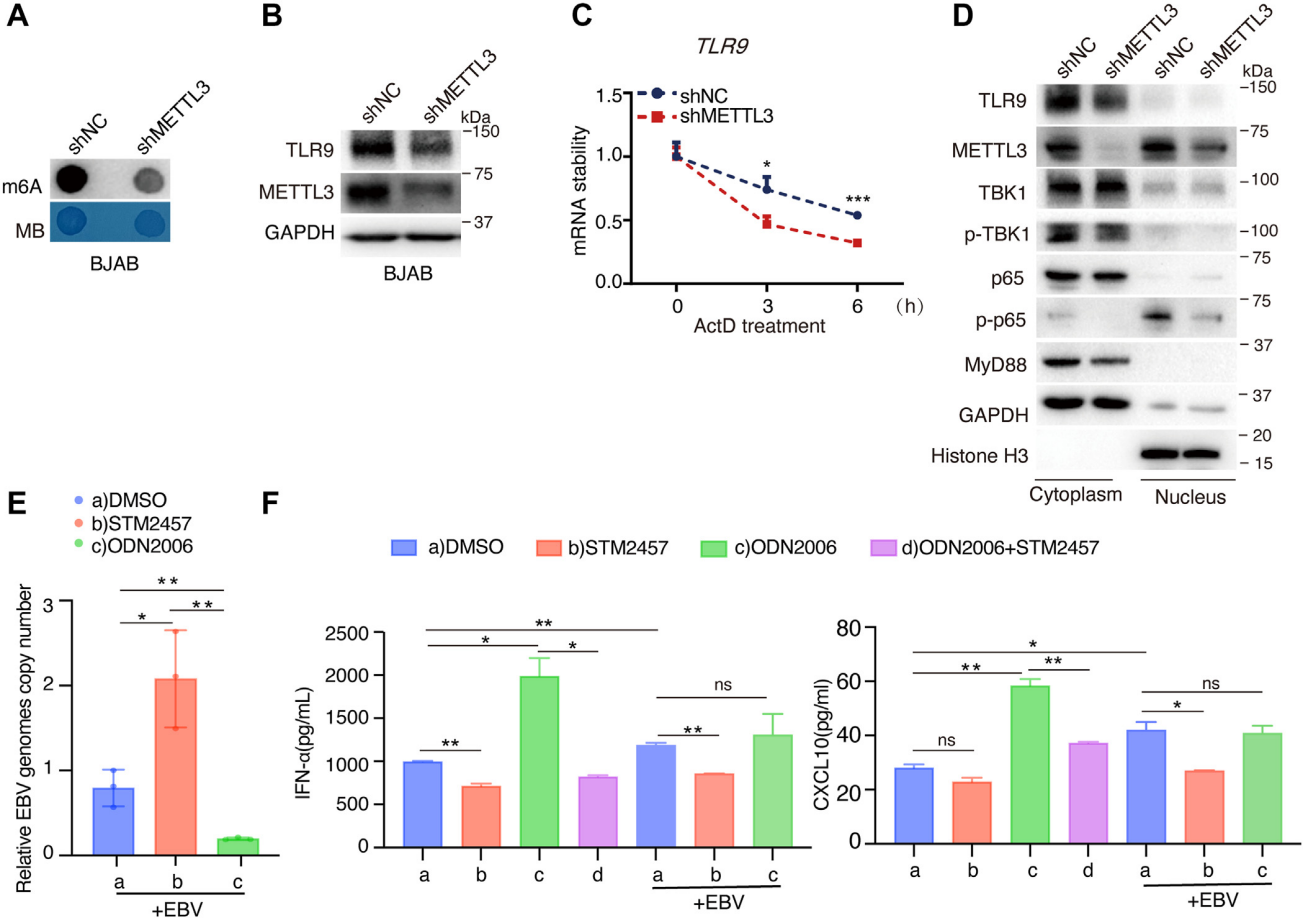
**TLR9 downstream signaling pathways and cytokine expression are regulated by METTL3.***A*, cellular m^6^A modification levels were assayed in BJAB-shNC and BJAB-shMETTL3 cells by Dot blot analysis. *B*, the expression levels of TLR9 and METTL3 were determined by Western blotting in BJAB-shNC and BJAB-shMETTL3 cells. *C*, BJAB-shNC and BJAB-shMETTL3 cells were treated with ActD (5 μg/ml) for 0, 3, and 6 h; TLR9 mRNA levels were measured by RT-qPCR. *D*, the indicated TLR9 signaling proteins were detected by cytoplasmic and nuclear proteins isolation. GAPDH and Histone H3 were used for cytoplasm and nucleus protein loading controls, respectively. *E*, BJAB cells were treated with STM2457(10 μM) and CpG-ODN2006 (5 μM) upon 50 MOI EBV infection, respectively. EBV genomes copy number was determined by qPCR. *F*, primary B cells were treated with STM2457(10 μM) and CpG-ODN2006(5 μM) with or without 50MOI EBV infection for appointed time. The secretion of IFN-α and CXCL10 were assayed by ELISA. These data are shown as the mean ± SD. ∗*p* < 0.05, ∗∗*p* < 0.01, ∗∗∗*p* < 0.001, ns, not significant. EBNA1, Epstein-Barr nuclear antigen 1; EBV, Epstein-Barr virus; IFN, interferon; m^6^A, N^6^-methyladenosine; MOI, multiplicity of infection; ODN, oligodeoxynucleotide; RT-qPCR, reverse transcription-quantitative PCR; TLR9, toll-like receptor 9.

TLR9, expressed predominantly in B-cells, is capable of being activated by unmethylated CpG (a motif found in bacterial and viral DNA) to initiate signaling pathways that regulate the production of proinflammatory cytokines or interferons (47). EBV infection suppresses TLR9 m^6^A modification levels which aroused our great interest, so we intended to explore further the underlying mechanism. We constructed a BJAB cell line with METLL3-shRNA inhibition. Since METLL3 is an m^6^A “writer”, the total cellular m^6^A levels were decreased upon METLL3 knockdown (Fig. 2*A*). Additionally, TLR9 protein levels were decreased following the knockdown of METTL3 (Fig. 2*B*). METTL3 is known to regulate RNA stability (13), and we explored whether METTL3 regulates TLR9 protein expression by affecting its mRNA stability. Using Actinomycin D to treat BJAB cells, we revealed that knockdown of METTL3 suppressed *TLR9* mRNA stability (Fig. 2*C*). The distribution of TLR9 signaling molecules was detected by nuclear and cytoplasmic protein extraction assay. METTL3 is localized in both the nucleus and the cytoplasm. TLR9 is localized mainly in the cytoplasm. TLR9 protein expression was inhibited by knockdown of METTL3, which inhibited phosphorylation levels of both TBK1 and p65. In addition, knockdown of METTL3 inhibited MYD88 protein expression (Fig. 2*D*). We utilized STM2457, a METTL3-specific inhibitor that reduces cellular m^6^A modification levels (48), and ODN2006 (5 μM), a specific TLR9 agonist, to assay their impact on EBV-positive cells. STM2457 could increase EBV copy numbers in cells (Fig. 2*E*), suggesting that METTL3 may inhibit EBV replication. EBV infection stimulated both IFN-α and CXCL10 expression in cells, whereas STM2457 could reverse this effect in EBV-positive primary B cells (Fig. 2*F*). Due to the fact that EBV infection significantly inhibited TLR9 expression, ODN2006 has little impact on IFN-α and CXCL10 expression in EBV-positive primary B cells (Fig. 2*F*).

### EBNA1 induces METTL3 degradation *via* the ubiquitin-proteasome pathway to inhibit host cellular m^6^A modification levels

**Figure 3:**
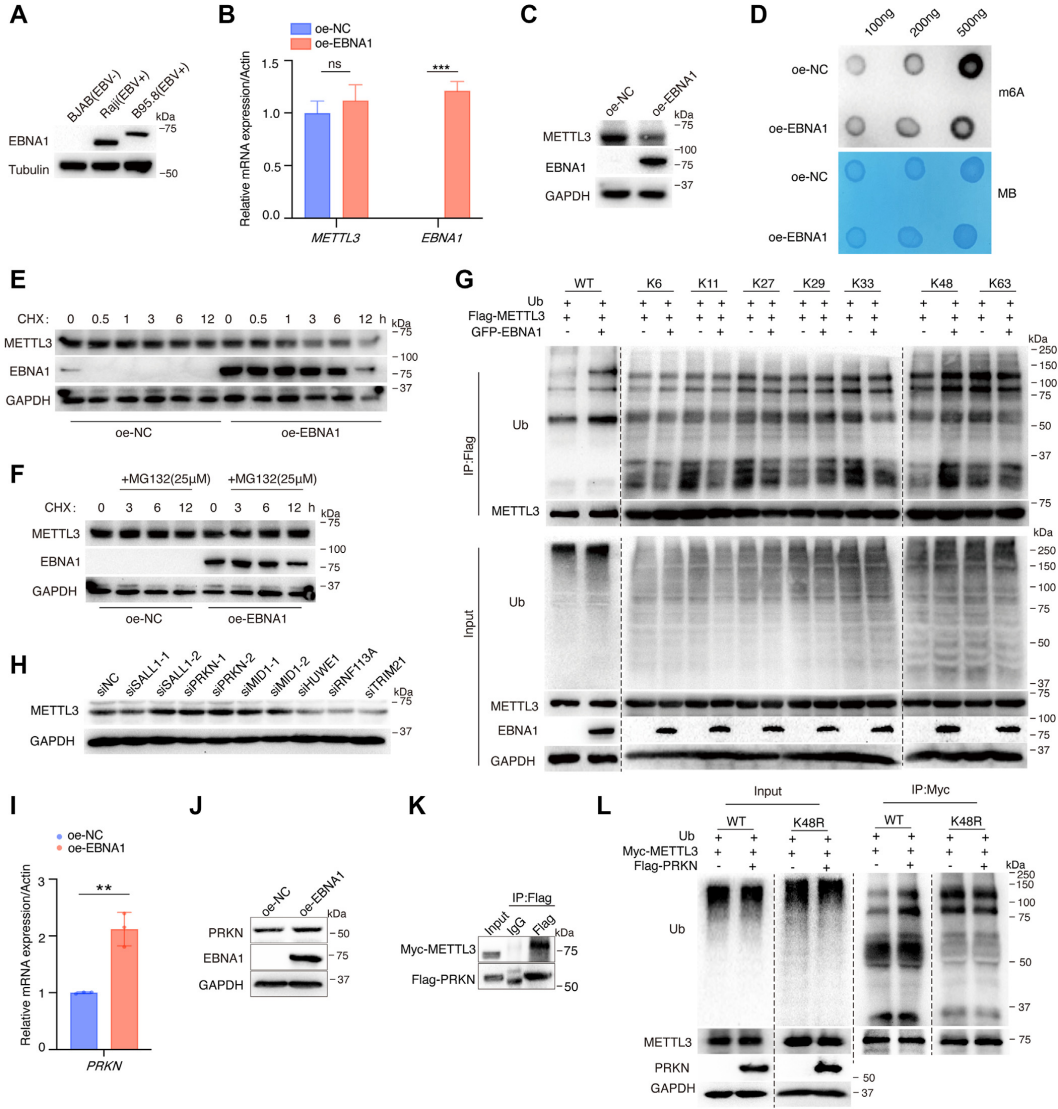
**EBNA1 induces METTL3 protein degradation *via* the ubiquitination pathway.***A*, EBNA1 protein levels were detected in BJAB, Raji, and B95.8 cells. *B* and *C*, Flag-NC and Flag-EBNA1 were transfected into HEK293 cells. The mRNA and protein levels of METTL3 and EBNA1 were assayed by RT-qPCR (*B*) and Western blotting (*C*). *D*, The 100 ng, 200 ng, and 500 ng mRNA from HEK293 cells transfected with Flag-EBNA1 (or Flag-NC as negative control) were extracted to detect the total cellular m^6^A modification level by Dot blot. A concentration of 0.02% methylene blue staining was used as a loading control. *E* and *F*, HEK293 cells were transfected with EBNA1 plasmids for 36 h, followed by CHX treatment (25 μg/ml) and MG132 (25 μM) for indicated times. Then cellular proteins were collected, and Western blotting was performed. *G*, GFP-NC and GFP-EBNA1, Flag-METTL3 and plasmids containing various polyubiquitin chains were cotransfected for 48 h. HEK293 cells were treated with MG132 (25 μM) for 4 h before harvest. Lysates were immunoprecipitated with anti-Flag beads. *H*, HONE-1 cells were transfected with indicated siRNAs for 48 h. The protein expression levels were detected by Western blotting. *I* and *J*, HONE-1 cells were transfected with indicated plasmids, then, the mRNA and protein expression of *PRKN* was measured by RT-qPCR (*I*) and Western blotting (*J*). *K*, HEK293 cells were transfected with Flag-PRKN and Myc-METTL3 plasmids for 48 h. The cell lysates were immunoprecipitated with anti-Flag beads (or IgG as control). The first lane was an input control without immunoprecipitation. *L*, Myc-METTL3, Flag-PRNK, and Ub (either WT or K48R) plasmids were cotransfected into HEK293 cells for 48 h. Cell lysates were immunoprecipitated with anti-Myc antibodies. The data are shown as the mean ± SD. ∗∗*p* < 0.01, ∗∗∗*p* < 0.001, ns, not significant. CHX, cycloheximide; EBNA1, Epstein-Barr nuclear antigen 1; m^6^A, N^6^-methyladenosine; RT-qPCR, reverse transcription-quantitative PCR.

We thus hypothesized that EBV inhibits TLR9 expression by modulating the host m^6^A modification system. EBNA1 was highly expressed in the EBV-positive cell lines Raji and B95.8 (Fig. 3*A*). Interestingly, TLR9 expression levels were negatively correlated with EBNA1 expression in BJAB and B95.8 cells (Fig. S2*A*). EBNA1 did not affect the METTL3 mRNA expression levels, but inhibited its protein levels (Fig. 3, *B* and *C*). Additionally, EBNA1 upregulated the protein levels of ALKBH5, but not FTO (Fig. S2*B*). Given that, we further examined the total cellular RNA m^6^A modification levels. m^6^A dot blot assay revealed that EBNA1 significantly reduced the cellular m^6^A levels (Fig. 3*D*). We utilized cycloheximide (CHX) to treat cells and found that EBNA1 promotes METTL3 protein degradation (Fig. 3*E*), whereas the proteasome inhibitor MG132 (25 μM) treatment reversed this effect (Fig. 3*F*). Moreover, to determine which type of ubiquitin linkage of polyubiquitin chains on METTL3 is mediated by EBNA1, we transfected plasmids of polyubiquitin chains conjugated to the lysine 6 site (K6), K11 site, K27 site, K29 site, K33 site, K48 site, and K63 site. Figure 3*G* shows that expression of EBNA1 increased K48-linked ubiquitination of METTL3 without affecting other types of linkages, indicating that EBNA1 primarily increases K48-linked polyubiquitin chains of METTL3. Next, we utilized UbiBrowser 2.0 (49) and mass spectrometric analysis result for METTL3 (50) to predict the E3 ubiquitin ligases that may regulate METTL3 protein degradation (Fig. S2, *C* and *D*). siRNAs targeting these candidate E3 ubiquitin ligases were transfected into cells, and a substantial increase in the protein levels of METTL3 were observed after inhibiting the expression of the E3 ligases SALL1, PRKN, and MID1 (Figs. 3*H* and S2, *E−H*). Thus, we proposed whether EBNA1 facilitates METTL3 ubiquitination degradation by regulating these E3 ligases. EBNA1 could upregulate the expression levels of PRKN (Fig. 3, *I* and *J* and S2*I*). In addition, coimmunoprecipitation (co-IP) demonstrated an interaction between PRKN and METTL3 (Fig. 3*K*). To test whether PRKN increased the K48-linked polyubiquitin chains of METTL3, we transfected Myc-METTL3 together with His-ubiquitin, or His-K48R ubiquitin mutant retaining all but one lysine residues (KR) in the presence or absence of PRKN followed by ubiquitination analysis. Consistent with these observations, PRKN catalyzed increase of K48-linked polyubiquitin chains from METTL3 in cells (Fig. 3*L*). This result suggests that EBNA1 increases METTL3 protein ubiquitin degradation, which mediated by the E3 ligase PRKN, and thus inhibits total m^6^A modification levels of host cells.

### TLR9 is an m^6^A-modified target gene of YTHDF1

**Figure 4:**
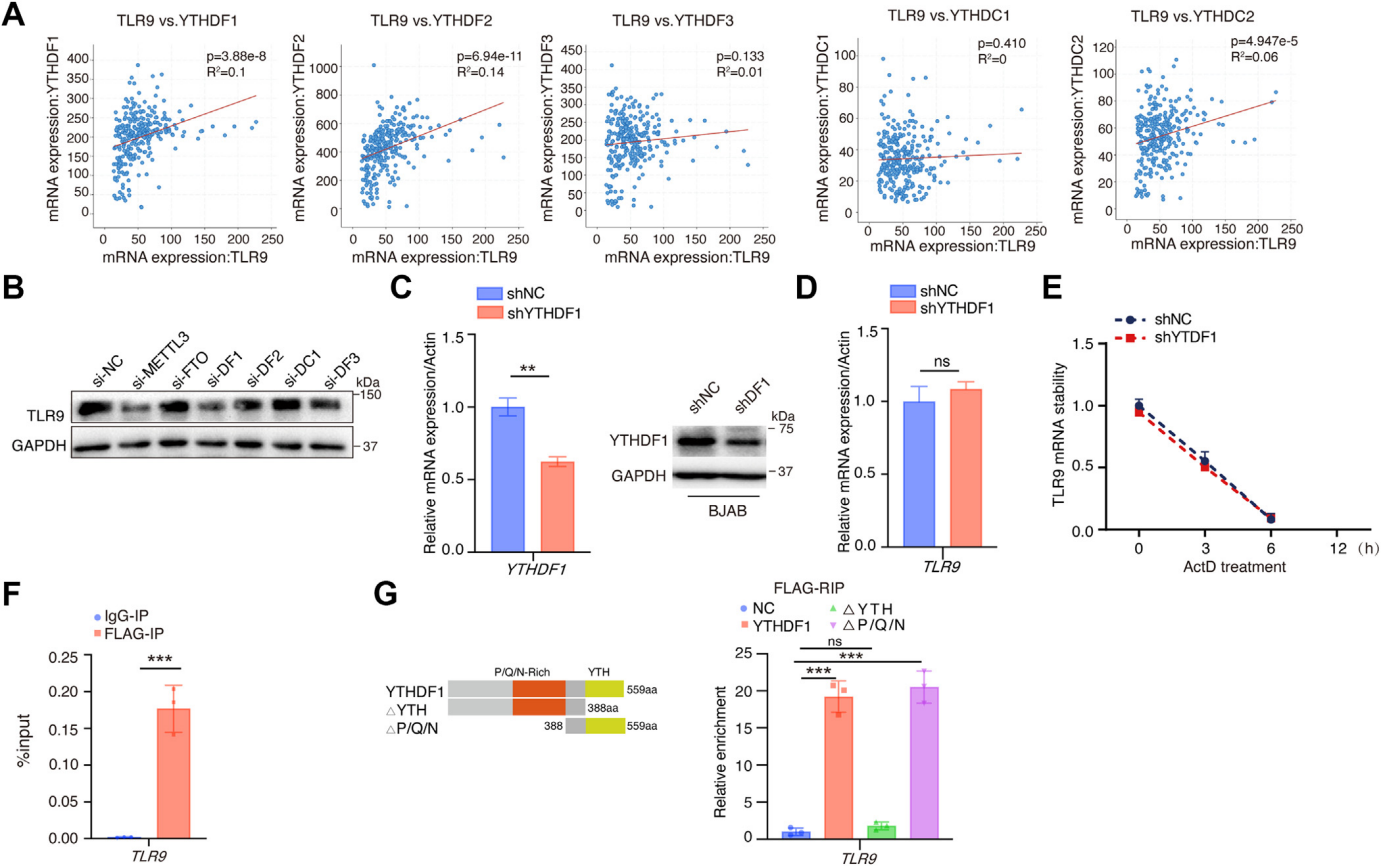
**YTHDF1 can bind TLR9 mRNA and inhibit itsprotein expression.***A*, cBio Cancer Genomics Portal (cBioPortal) website analyzes m^6^A readers and TLR9 correlation in mature B-cell malignant lymphomas. *B*, BJAB cells were transfected with indicated siRNAs for 48 h to assay their impact on TLR9 protein expressions. *C* and *D*, BJAB-shNC and BJAB-shYTHDF1 were constructed by lentiviral infection and screened by puromycin to a certain time. The expression levels of YTHDF1 and TLR9 were detected by RT-qPCR and Western blotting. *E*, BJAB-shNC and BJAB-shYTHDF1 cells were collected to examine *TLR9* mRNA stability using ActD (5 μg/ml) treatment for indicated time. *F*, HEK293 cells were cotransfected with HA-TLR9 and Flag-YTHDF1 plasmids for RNA immunoprecipitation experiments, and the *TLR9* mRNA levels from the immunoprecipitated RNAs were assayed by RT-qPCR. *G*, schematic representation of YTHDF1 structural domain and indicated deletion mutants (*left*). The full or the mutant YTHDF1 plasmids were transfected into HEK293 cells to detect the combination with *TLR9* mRNA. RNA immunoprecipitation was performed using normal mouse IgG or anti-Flag antibody. IgG precipitated RNA enrichment was used as the control. Data are presented as mean ± SD from three independent experiments. ∗∗*p* < 0.01*,* ∗∗∗*p* < 0.001, ns, not significant. IgG, immunoglobulin G; m^6^A, N^6^-methyladenosine; RT-qPCR, reverse transcription-quantitative PCR; TLR9, toll-like receptor 9.

Mechanism that regulates TLR9 expression through m^6^A modification was explored in depth. First, we used the cBio Cancer Genomics Portal (cBioPortal, http://cbioportal.org) (51) to analyze whether TLR9 correlates with m^6^A “readers” expression in mature B-cell malignancies. In 760 samples of human mature B-cell malignancies, TLR9 expressions are positively correlated with the expressions of YTHDF1, YTHDF2, and YTHDC2, but not YTHDF3 and YTHDC1 (Fig. 4*A*). In BJAB cells, siRNAs targeting YTHDF1 as well as METTL3 induced a reduction of TLR9 expressions (Fig. 4*B* and S3*A*). To further examine how m^6^A “reader” YTHDF1 regulates TLR9 expression through m^6^A modification, we used lentivirus shRNA to knockdown YTHDF1 in BJAB cells (Fig. 4*C*), and found that knockdown of YTHDF1 did not affect *TLR9* mRNA expression levels (Fig. 4*D*) as well as *TLR9* mRNA stability (Fig. 4*E*). As an m^6^A “reader” to regulate translation, YTHDF1 must bind to its target RNA. RIP-qPCR experiments confirmed that YTHDF1 binds to *TLR9* mRNA (Fig. 4*F*), suggesting that m^6^A -modified TLR9 may be a target of the m^6^A “reader” YTHDF1. We generated plasmids expressing truncated YTHDF1 (Fig. S3*B*), and found that deletion of the YTH structural domain disrupted the YTHDF1-TLR9 binding, whereas deletion of the pro/Gln/Asn-rich structural domain (P/Q/N) had no impact on this binding (Fig. 4*G*). These results indicate that YTHDF1 protein binds to *TLR9* mRNA, and the YTH domain is required to mediate this interaction.

### YTHDF1 regulates TLR9 translation in an m^6^A-dependent manner

**Figure 5:**
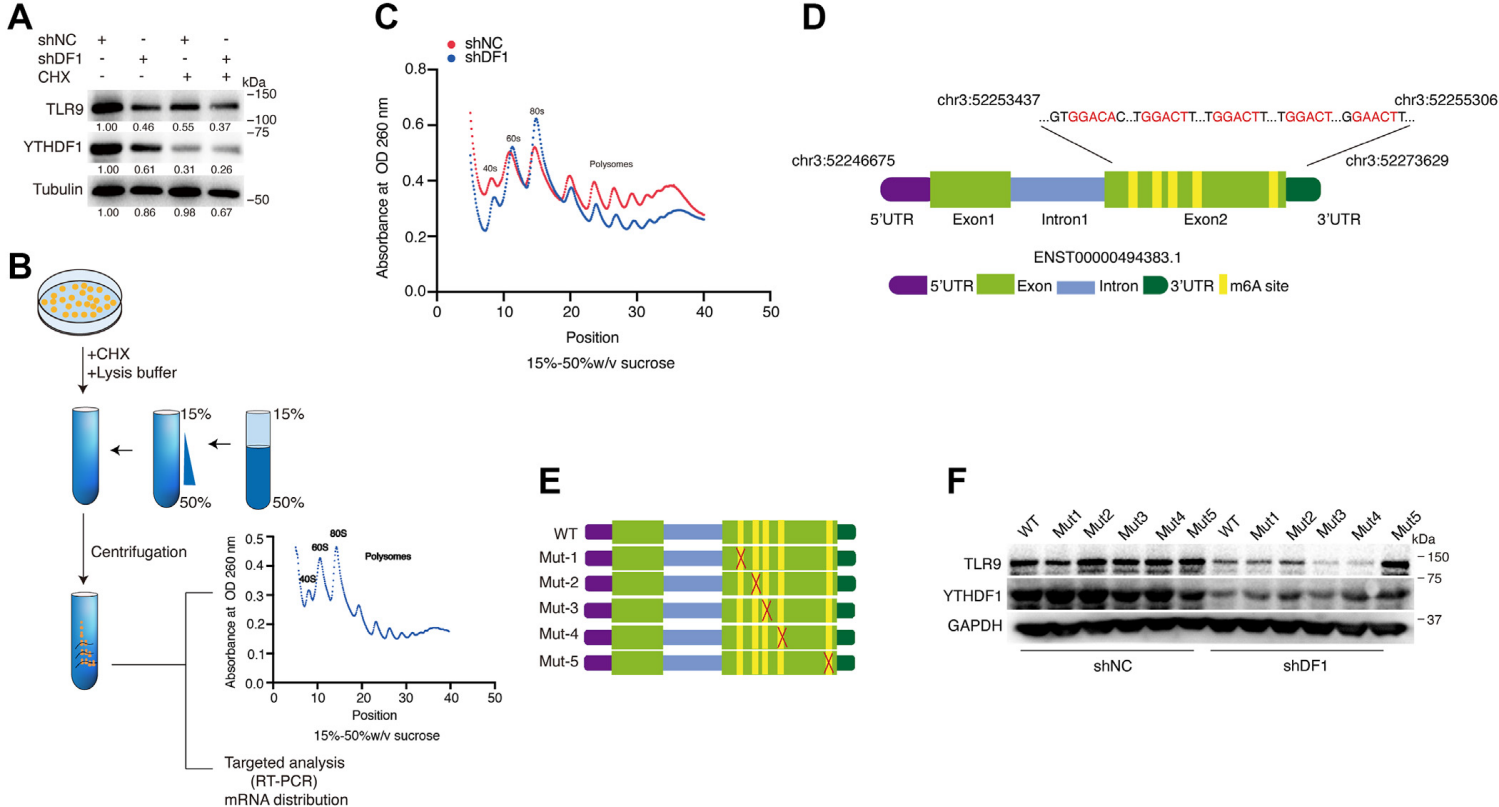
**Knockdown of YTHDF1 inhibits TLR9 mRNA translation *via* m**^**6**^**A modification manner.***A*, BJAB-shNC and BJAB-shDF1 cells were treated with CHX (25 μg/ml) for 4 h before harvest. The indicated proteins expression levels were detected. *B*, the schematic diagram illustrated the ribosome profiling analysis. *C*, ribosome profiling was performed in HEK293-shNC and HEK293-shDF1 cells. *D*, the schematic diagram of m^6^A motif position within TLR9 transcript. *E*, schematic representation of mutated m^6^A sites (GGAC to GGTC) of pcDNA3.1-3xFlag vector to investigate the m^6^A roles on TLR9 expressions. *F*, WT TLR9 and TLR9-Mut1-5 were transfected into HEK293 cells for 48 h. Protein expression was measured by Western blotting. CHX, cycloheximide; m^6^A, N^6^-methyladenosine; TLR9, toll-like receptor 9.

It has been revealed that YTHDF1 binds m^6^A-modified mRNAs and increases translational output through interactions with initiation factors and ribosomes (14). We hypothesized that the downregulation of TLR9 protein levels is due to YTHDF1 affecting translational efficiency. To verify this, we treated BJAB cells (sh-NC *versus* sh-YTHDF1) with the protein translation inhibitor CHX. Knockdown of YTHDF1 observably inhibited TLR9 protein expression levels, and the rate of TLR9 protein degradation was reduced with the addition of CHX (Fig. 5*A*). We used sucrose density gradient centrifugation to separate the cellular RNA fractions: nontranslating fraction (<40S), translational initiation fraction (including 40S ribosomes, 60S ribosomes, 80S monosomes, and <80S), and translationally active polysomes (>80S), for ribosome profiling analysis (Fig. 5*B*). A significant reduction in the polysome fraction (>80S) was observed in sh-YTHDF1 cells (Fig. 5*C*), suggesting that YTHDF1 increased the translational output of TLR9 mRNA.

Sequence-based RNA adenosine methylation site predictor website (52) combined with MeRIP sequencing results identified the presence of five m^6^A modification sites in *TLR9* mRNA, demonstrated in Figure 5*D*. We constructed a series of mutant TLR9 vectors (Mut-1 to Mut-5) (Figs. 5*E* and S4*A*) by mutating the potential m^6^A motif (“A” to “T”) and then transfected into cells (sh-NC *versus* sh-YTHDF1). Knockdown of YTHDF1 inhibited TLR9 protein expression levels, whereas Mut-5 could reverse this impact. We next performed RIP and found that Mut-5 did not bind YTHDF1 (Fig. S4*B*). These results indicate that m^6^A modification plays an important regulatory role at the site 5 of TLR9 mRNA.

### METTL3 inhibitor STM2457 suppresses TLR9-induced B cell proliferation and Ig antibody secretion

**Figure 6:**
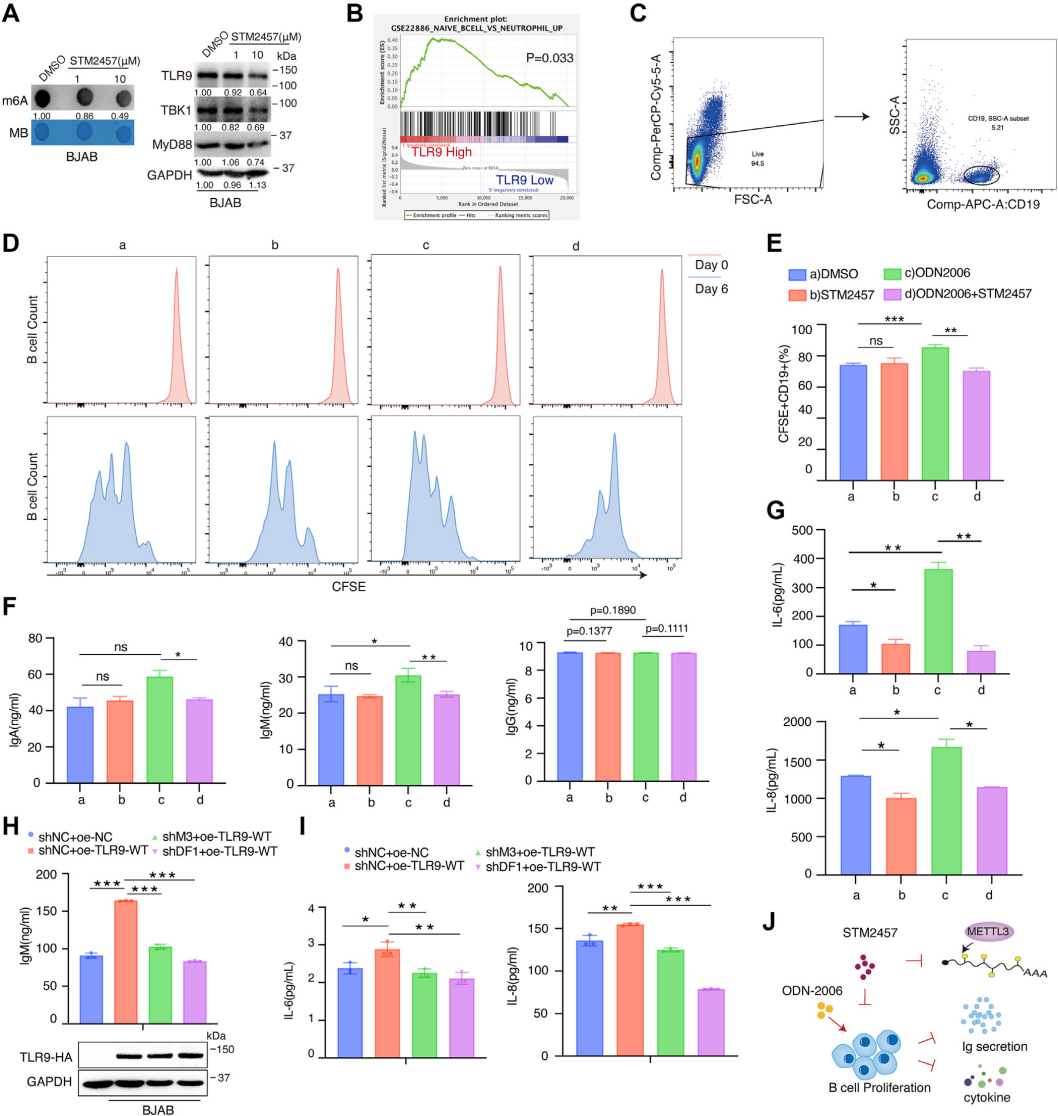
**STM2457 inhibits CpG-ODN-induced B cell proliferation, Ig secretion and cytokine expressions.***A*, BJAB cells were treated with STM2457 for 96 h. Cellular m^6^A modification levels were assayed by dot blot analysis. TLR9 signaling molecules were assayed by Western blotting. *B*, gene set difference between the TLR9 high and low human immune cells was revealed by GSEA. The top gene set enriched in TLR9 high group comparing to low group is listed. *C–E*, PBMCs from healthy donors were stained with CFSE dyes and cultured with anti-IgM (10 mM), CD40L (1 mM), and IL-4 (10 nM), costimulated with STM2457(10 μM) or ODN-2006(5 μM) for 0 and 6 days. DMSO was used as a control. Only CD19+ B cells stained CFSE dyes were gated by flow cytometry analysis (*C* and *D*). *E*, histogram shows the percentage of B cells dividing under different conditions. *F* and *G*, fresh PBMCs cultured with anti-IgM, CD40L, IL-4, and costimulated with STM2457(10 μM) or ODN-2006(5 μM) for 8 days were collected to detect Ig-producing cells (*F*) and cytokine level in supernatants by ELISA analysis (*G*). *H* and *I*, BJAB-shRNA cells were transfected with HA-TLR9 plasmids for 48 h. Then, the levels of Ig (*H*) and cytokine (*I*) in supernatants were detected by ELISA analysis. *J*, model diagram demonstrates the role of STM2457 in the regulation of B-cell proliferation and cytokines secretion. Experiments were independently repeated three times, and results are presented as mean ± SD. ∗*p* < 0.05, ∗∗*p* < 0.01, ∗∗∗*p* < 0.001, ns, not significant, compared with the control group. CFSE, carboxyfluorescein succinimidyl ester; DMSO, dimethylsulfoxide; GSEA, gene set enrichment analysis; Ig, immunoglobulin; IL, interleukin; m^6^A, N^6^-methyladenosine; ODN, oligodeoxynucleotide; PBMC, peripheral blood mononuclear cell; TLR9, toll-like receptor 9.

STM2457 is a highly potent and catalytic inhibitor of METTL3. Dot blot analysis showed that STM2457 (10 μM) inhibited total cellular m^6^A levels in BJAB cells (Fig. 6*A*). To our surprise, STM2457 upregulated ALKBH5 protein expression, but not FTO and YTHDF1 (Fig. S5*A*). In addition, STM2457 (10 μM) inhibited the protein levels of TLR9 and the downstream signaling molecules TBK1 and MYD88 (Fig. 6*A*). TLR9 recognizes bacterial or viral DNA sequences in an unmethylated CpG motif. In humans, TLR9 expression is restricted to pDCs and B lymphocytes (53). Gene set enrichment analysis (GSEA) revealed that clinical specimens with TLR9-high expression were enriched in B cells compared to TLR9-low expression specimens (Figs. 6*B* and S5*B*). In B cells, TLR9 activation drives costimulatory molecules expression, cell survival and proliferation, IL-6 and IL-10 production, terminal differentiation, and Ig secretion (54, 55). We thus explored whether STM2457 affects TLR9-induced proliferation and Ig antibody secretion in primary B cells. Fresh peripheral blood mononuclear cells (PBMCs) were extracted from healthy human peripheral blood treated with anti-BCR (B-cell-receptor) antibody and CD40L for 6 days in the presence or absence of CpG-ODN. Primary B cells were isolated from PBMCs using anti-human CD19-APC Abs (Fig. 6*C*). B cell proliferation was assayed by proliferation labeling with carboxyfluorescein succinimidyl ester (CFSE) dye. Flow cytometry demonstrated that ODN2006, a TLR9 agonist, promoted B cell proliferation, and STM2457 inhibited the ODN-2006-induced B cell proliferation (Fig. 6, *D* and *E*). Next, we examined the effect of STM2457 on Ig secretion from TLR9-stimulated B cells. Under CpG-ODN stimulation, ODN2006 stimulated IgA and IgM secretion in human B cells, whereas STM2457 reversed this effect (Fig. 6*F*). ODN2006-induced IL-6 and IL-8 secretions were reduced in the presence of STM2457 (Fig. 6*G*). To further examine whether changes in Ig antibodies and cytokines production are caused by altered TLR9 m^6^A modification, we transfected plasmids including HA-TLR9-WT and HA-TLR9-Mut to verify this hypothesis. Expression of HA-TLR9-WT promoted IgM, IL-6, and IL-8 secretion, whereas knockdown of METTL3 or YTHDF1 significantly reduced their production levels in supernatants from BJAB cells (Figs. 6, *H* and *I* and S5, *E* and *F*). Consistently, ELISA results revealed that the production of IgM and IL-8 was enhanced with the HA-TLR9-WT plasmid, but not the mutant site 5 in BJAB cells (Fig. S5, *C* and *D*). To summarize, we found that STM2457, an METTL3 inhibitor, could suppress TLR9 expression and its immune-stimulation functions. This might be explained by the fact that STM2457 inhibits METTL3 activity leading to reduced TLR9 m^6^A modification (Fig. 6*J*).

### STM2457 antagonizes TLR9 function to assist immune escape of certain tumor cells

**Figure 7:**
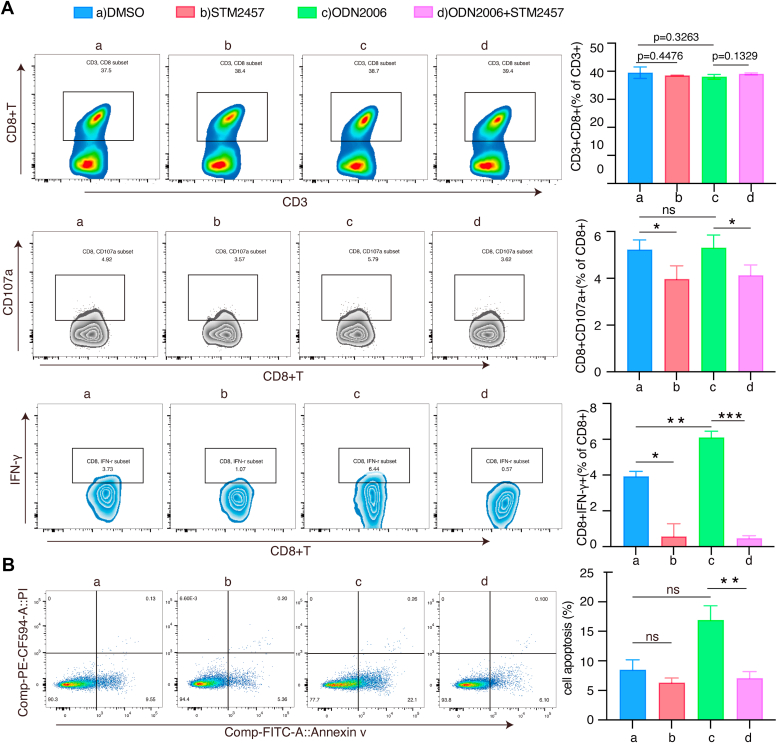
**METTL3 inhibitor STM2457 suppresses CpG-ODN-induced antitumor immunity.** Primary T-cells were stimulated with IL-2 (50 ng/ml) and ImmunoCult Human CD3/CD28/CD2 T Cell Activator (25 μl/ml). T cells were cocultured with BJAB cells (T cells: BJAB cells ratio=8:1) under different condition. Total cells were treated with cell activation cocktail (with Brefeldin A) for 6 h before collected, then, detected by flow cytometry. *A*, representative histograms of CD8^+^ T cells, CD8^+^ CD107a^+^ T cells, and CD8^+^IFN-γ^+^T cells were shown. Quantitation of each group was shown in the right panels. *B*, the proportion of BJAB cells undergoing apoptosis were detected by flow cytometry. The results are presented as mean ± SD. ∗*p* < 0.05, ∗∗*p* < 0.01, ∗∗∗*p* < 0.001, ns, not significant. IFN, interferon; ODN, oligodeoxynucleotide.

TLR9 agonist CpG ODN has been recognized as a promising adjuvant for cancer vaccines. It enhances the antitumor effects of T cells, shifting the immune response toward a Th1 phenotype (56, 57). We further explored whether STM2457 could influence TLR9 function in antitumor immunity. Primary T cells from PBMC extracted from healthy donors were cocultured with BJAB cells (T cells: BJAB cells = 8:1) (58). STM2457 did not affect the number of CD8^+^ T cells, but inhibited the expression levels of CD107a^+^, IFN-γ^+^, and TNF-α^+^ in CD8^+^ T cells. Treatment of cocultured cells with ODN2006 remarkably promoted the expression levels of IFN-γ^+^ in CD8^+^ T cells, as well as the expression levels of CD107a^+^ and IFN-γ^+^ in CD4^+^ T cells. The expression levels of CD107a^+^ and IFN-γ^+^ in CD8^+^ T and CD4^+^ T cells were inhibited when cocultured cells were treated with ODN2006 coupled with STM2457 (Fig. 7*A* and S6, *A* and *B*). Further, STM2457 inhibited apoptosis of BJAB cells in the coculture system (Fig. 7*B*). These results demonstrate that inhibition of cellular m^6^A levels could repress TLR9-induced antitumor immunity and assist tumor cell immune escape.

### Expressions of METTL3, YTHDF1, and TLR9 are highly correlated with immune cells infiltration in clinical samples

**Figure 8:**
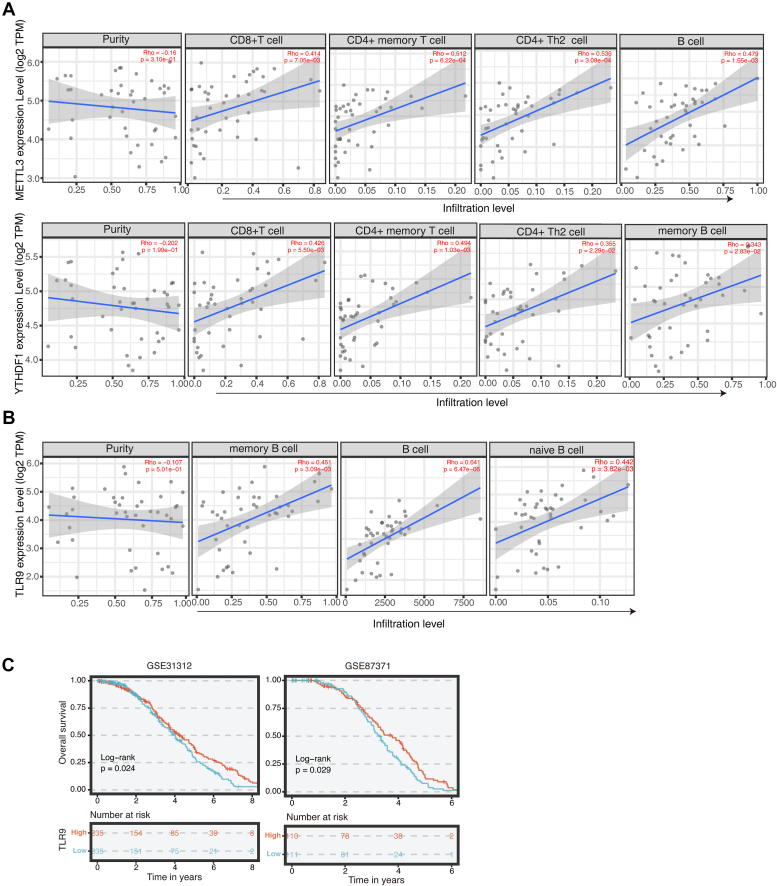
**METTL3, YTHDF1, and TLR9 are highly correlated with immune cell infiltration in B lymphomas samples.***A* and *B* Timer 2.0 was used to analyze the positive correlation between METTL3, YTHDF1, and TLR9 and immune infiltration in diffuse large B lymphomas, including CD8^+^ T cells, CD4^+^ T cells, and B cells. *C*, Kaplan–Meier plots of the relapse-free survival of diffuse large B cell lymphoma (DLBCL) patients. Patients were stratified with high (*red*) or low (*blue*) expression of TLR9. TLR9, toll-like receptor 9.

In general, cytotoxic T cells, memory T cells, Th1 cells, Tfh cells, and B cells are associated with prolonged survival, whereas elevated density of Treg cells, myeloid-derived suppressor cell and neutrophils are associated with poor prognosis (59). We analyzed the correlation of METTL3, YTHDF1, and TLR9 and immune infiltration in diffuse large B-cell lymphomas including CD8^+^ T cells, CD4^+^ T cells, and B cells from The Cancer Genome Atlas data *via* Timer 2.0 (http://timer.cistrome.org/). Combined analysis by xCell, MCP-counter, CIBERSORT, EPIC, and other tools revealed that the expressions of METTL3 and YTHDF1 had a strong positive correlation with CD8^+^ T cells, CD4^+^ T cells, and B cells in tumors (Fig. 8*A*). This implies a possibility that METTL3 and YTHDF1 could affect the immune inflammatory response and immune infiltration. Besides that, TLR9 expression was significantly positively correlated with B cells in diffuse large B-cell lymphomas (Fig. 8*B*). Meanwhile, we analyzed 760 samples of B-cell malignant tumors, and the survival rates of patients with high TLR9 expression were all better than that of patients with low expression, suggesting a close relationship between high TLR9 expression and a good prognosis in patients with B-cell lymphoma (Fig. 8*C*). This analysis suggests that METTL3, YTHDF1, and TLR9 may have influence on lymphoma development and prognosis, *via* modulating immune microenvironments.

## Discussion

**Figure 9:**
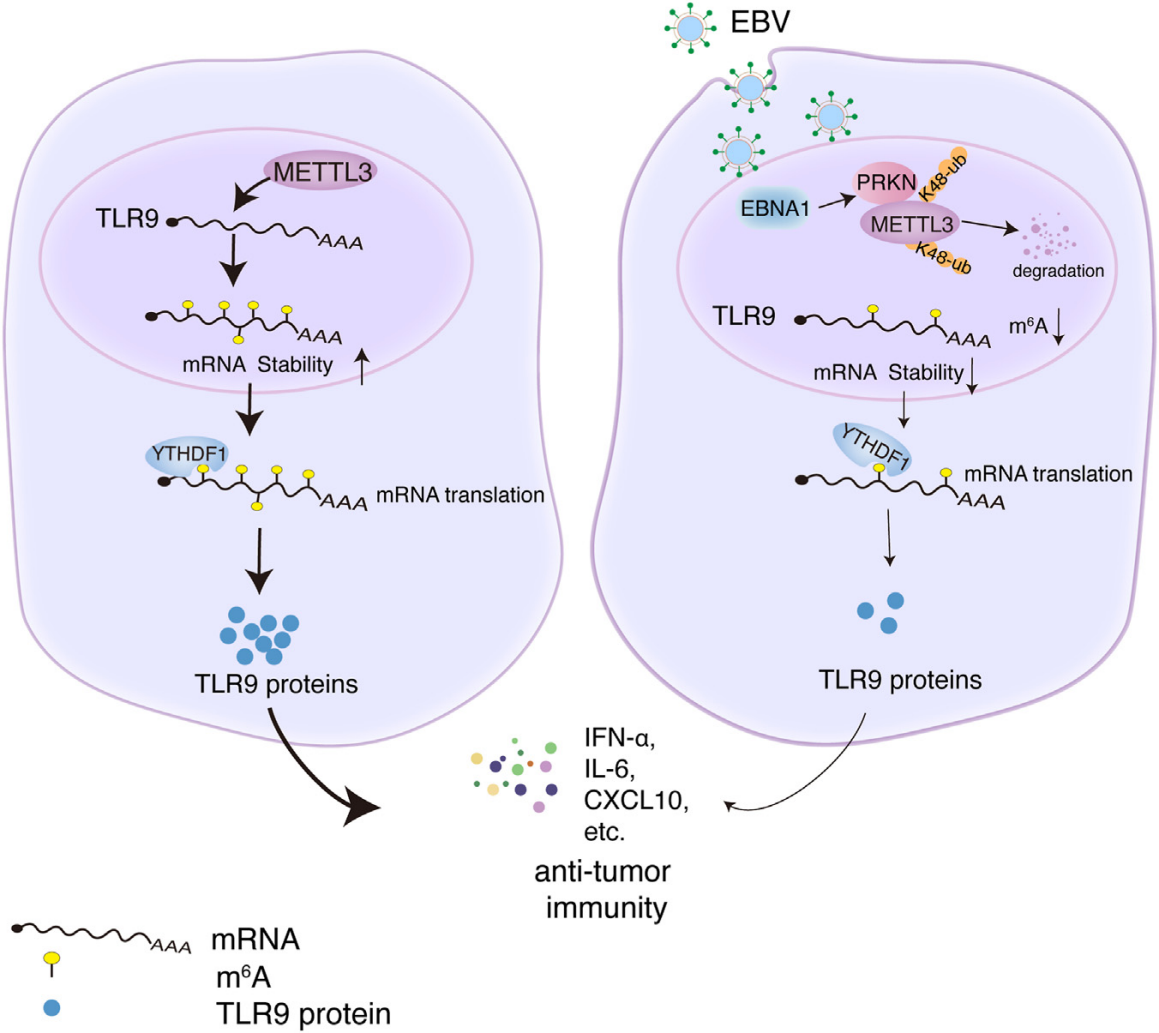
**A working model depicting the main molecular mechanisms about the current study.** EBV EBNA1 increases METTL3 protein degradation *via* K48-linked ubiquitin-proteasome pathway, which mediated by the E3 ligase PRKN, thus inhibits cellular m^6^A modification. Downregulation of METTL3 inhibits TLR9 m^6^A modification. YTHDF1 is an m^6^A “reader” for cellular *TLR9* mRNA with m^6^A modification, and YTHDF1 binding increases *TLR9* mRNA translation efficiency. EBV infection decreases the m^6^A levels of *TLR9* mRNA, thus reduces TLR9 protein levels, as well as TLR9-induced cytokine secretions. EBV, Epstein-Barr virus; m^6^A, N^6^-methyladenosine; TLR9, toll-like receptor 9.

m^6^A methylation modifications are presented in DNA viral infections and life cycles, including herpes simplex virus-1 (HSV-1) (60), HBV (61), Kaposi's sarcoma-associated herpesvirus (KSHV) (62, 63), EBV (28, 41, 42, 64). Our previous study showed that EBV infection alters the level and distribution of m^6^A modifications in host cell transcripts (43). Interestingly, we found that EBV significantly inhibited TLR9 m^6^A modification and expression levels. Meanwhile, we found for the first time that the EBV-encoded nuclear antigen EBNA1 increases METTL3 protein degradation *via* K48-linked ubiquitin-proteasome pathway, which mediated by the E3 ligase PRKN, resulting in reduced levels of cellular m^6^A modification. Owing to the essential role of TLR9 in innate and antitumor immunity, we explored how m^6^A modification regulates TLR9 function. We revealed that METTL3 promotes TLR9-induced B cell proliferation and Ig antibody secretion, as well as CpG-ODN-induced antitumor immunity. Thus, we reveal a novel mechanism that indicates the m^6^A modifications involvement in EBV-host-mediated antitumor immunity (Fig. 9).

EBNA1 is the only viral protein required for EBV replication in latently infected cells, and it is presented in all EBV-related malignancies (65). EBNA1 has multiple functions that maintain EBV episome replication and regulate latency transcription (66, 67, 68, 69). In this study, we showed that EBNA1 can reduce the overall m^6^A modification level in host cells by inhibiting METTL3 expression through K48-linked ubiquitination pathway. This suggests that EBNA1 can regulate host gene expression partly by altering host cell m^6^A modification levels. In addition, targeting EBNA1 or other latent genes has important research significance for the treatment of EBV-associated malignancies because it can be used as a marker to distinguish EBV-associated cancer cells from normal cells.

The methyltransferases METTL3, METTL14, and WTAP works as a complex to write m^6^A modifications on eukaryotic mRNAs (70). m^6^A-modified residues can be recognized by YTH family proteins, which direct the mRNA to the cell membrane compartment to undergo stabilization or degradation (71). YTHDF1 and YTHDF3 are known to promote protein synthesis by regulating mRNA translation (14, 72, 73); YTHDF2 recognizes m^6^A sites to regulate mRNA stability and mRNA degradation (13). Depletion of METTL3-METTL14 and its accessory subunits WTAP and ZC3H13 resulted in an increase in the mRNA abundance of intracisternal A-particles and related ERVK elements (74). Knockdown of METTL3 significantly abrogated PD-L1 m^6^A modification and decreased *PD-L1* mRNA stability (75). We identified that METTL3 increases *TLR9* mRNA stability and protein expression. YTHDF1 increases TLR9 protein expression by regulating its mRNA translation. This suggests that m^6^A modification promotes host gene *TLR9* stabilization and expression.

TLR9 recognizes bacterial or viral DNA to trigger an innate immune response. HBV infection inhibited TLR9 expression in human primary B cells and impaired TLR9-mediated B cell proliferation and secretion of the pro-inflammatory cytokine IL-6, but did not affect total Ig antibody secretion (76). HBV particle internalization inhibited TLR9-mediated IFN-α secretion from pDCs and TLR9 transcriptional activity in B cells (77). The viral dsDNA sensor TLR9 is expressed on B cells, which are natural targets for EBV infection. TLR9 has been shown to inhibit EBV lytic gene BZLF1 transcription through histone modification in Burkitt's lymphoma cells (78). Additionally, TLR9 recognizes EBV in a different manner in primary monocytes and pDC for coordinated antiviral immunity (79). The reason for this may be the differences in the period of infection and cell type. The current study presents for the first time that EBV regulates TLR9 expression through m^6^A modification in human Burkitt's lymphoma cells. The methyltransferase METTL3 increases TLR9 expression levels by promoting mRNA stabilization. According to our results, METTL3 inhibitor STM2457 suppressed CpG-ODN-induced B cells proliferation, Ig secretion, and cytokine IL-6/IL-8 expression. Molinari *et al.* revealed that Autographa californica multiple nuclear polyhedrosis virus (AcMNPV) carrying a fragment of OVA (chicken egg ovalbumin) fused to the N terminus of VP39 protein (BV-cOVA) triggers endosomal TLR9 signaling in DCs, which triggers a CD8^+^ T cell-mediated adaptive immune response through the TLR9/MyD88 pathway (80). We identified that STM2457 treatment in the cocultured cell systems can inhibit the expression levels of CD107a^+^ and IFN-γ^+^ in CD8^+^ T and CD4^+^ T cells upon CpG-ODN stimulation. These results suggest that inhibition of cellular m^6^A levels can reverse TLR9-induced antitumor immunity and assist in tumor cell immune escaping. m^6^A modification may be an important mechanism in regulating TLR9 expression and functions.

In summary, we reported that the EBV infection suppressed TLR9 expression through m^6^A mechanism. EBV EBNA1 promotes METTL3 degradation, which leads to a decrease in cellular m^6^A modification levels. Suppression of m^6^A modification levels inhibited TLR9 expression and its antitumor immunity function. Our study revealed a novel mechanism by which EBV utilizes the m^6^A modification system to regulate innate immune molecules to promote immune evasion. This discovery deepens our understanding of the critical role of m^6^A modification in oncogenic virus-host interactions, and offers a possibility of developing a novel therapeutic strategy for modulating cellular m^6^A modifications contributing to the therapy of EBV-associated tumors.

## Experimental procedures

### Cell culture and reagents

BJAB (EBV-negative Burkitt's lymphoma cell line), Raji (EBV-positive Burkitt's lymphoma cell line), B95.8 (EBV-transformed tamarin (*Saguinus oedipus*) cell line) cells and HONE-1 (human nasopharyngeal carcinoma cell line) were cultured in RPMI 1640 supplemented with 10% fetal bovine serum (FBS). HEK293 cells were cultured in Dulbecco’s modified Eagle medium supplemented with 10% FBS. All cells were confirmed to be *Mycoplasma* negative before culture (TaKaRa) and were authenticated by the short tandem repeat Multiamplification Kit (Goldeneye DNA ID system 20A, Peoplespot) every six months. PBMCs were isolated from whole blood of healthy donors by Ficoll centrifugation. Primary B cells were isolated from PBMCs by MojoSort human Pan B cell Isolation Kits (480081, BioLegend) and were cultured in RPMI 1640 medium supplemented with 10% FBS. All cell lines were grown in a humidified incubator at 37 °C with 5% CO_2_ and obtained from American Type Culture Collection. CHX (M4879) were purchased from AbMole BioScience. STM2457 (HY-134836) and MG132 (HY-13259) was purchased from MedChemExpress.

### EB virus preparation and infection

B95.8 cells were expanded and cultured in RPMI 1640 medium containing 10% FBS. Cells were centrifuged and started at a new culture at 2 × 10^5^/ml density in RPMI-1640 medium containing 2% FBS. The medium was not changed in this process. Centrifugation was performed at 3000*g* to remove cell sediments and debris. The cells were passed through a 0.45 μm Millipore filter, then further centrifuged at 50,000*g* at 4 °C, and resuspended in fresh FBS-free RPMI-1640 medium. We used a DNA Quantitative Fluorescence Diagnostic Kit (Sansure Biotech) to determine the multiplicity of infection (MOI) of EBV according to the manufacturer's protocol. A total of 5 × 10^5^ BJAB cells were infected with 50 MOI EBV for 24 or 48 h at 37 °C with 5% CO_2_. Cells were collected to extract cellular RNA or protein for RT-qPCR and Western blotting analysis.

### mRNA stability

BJAB-shNC and BJAB-shMETTL3 cells were treated with ActD (5 μg/ml, M4881, AbMole BioScience) for 0, 3, and 6 h. After the treatment, cells were collected, and RNA was extracted for RT-qPCR after the processing time.

### Cell transfection

Cells were cultured to a predetermined time. The siRNAs were transfected using Lipofectamine 3000 (Invitrogen, L3000015) according to the manufacturer's instruction. ShRNA lentiviruses (targeting METTL3 and YTHDF1) were obtained from GenePharma. Lentiviruses were transduced into cells according to the manufacturer's instructions. The siRNA and shRNA sequences are shown in Table S1.

### m^6^A-immunoprecipitation (RIP)

According to the manufacturer's protocol, intact poly-A-purified RNA was isolated using Magnetic mRNA Isolation Kit (S1550S, New England Biolabs). With reverse transcription with Oligo(dT) and PCR amplification, mRNA incubated with 5 μg m^6^A antibody (202003; Synaptic Systems) in IP buffer (pH 7.4) containing RNase inhibitor (N2111SV, Promega), for 2 h at 4 °C. Protein A/G magnetic beads (823202, Selleck) were washed, incubated with mixture buffer for 2 h at 4 °C with rotation. m^6^A RNA was eluted with 6.7 mM m^6^A sodium salt (Santa Cruz Biotechnology) and precipitated with 100% ethanol. Enrichment of m^6^A was detected through RT-qPCR. Normal rabbit IgG were designed as negative control in this experiment group.

### Plasmid construction

EBNA1 plasmid is purchased from Addgene (#37954). DNA fragments encoding Flag-YTHDF1 and Flag-TLR9 were generated by PCR and cloned into a pcDNA3.1-3xFlag empty vector. pcDNA3.1-HA-TLR9 mut1 to mut5, pcDNA3.1-3xFlag-YTH mutant, and pcDNA3.1-3xFlag-P/Q/N mutant plasmids were synthesized (GeneScript). All plasmids were verified by sequencing.

### Immunoprecipitation and Western blotting

Cells were cultured with RPMI 1640 or Dulbecco’s modified Eagle medium supplemented with 10% FBS. After cells were harvested, the cell lysates were collected in IP lysis buffer (10 mM Tris, 1% NP-40, 2 mM EDTA, 150 mM NaCl [pH 7.5]) with Protease/Phosphatase Inhibitor Cocktail (B14001, B15001, Selleck). For IP, lysates were incubated with anti-Flag antibody (#F1804; Sigma-Aldrich) and anti-Mouse IgG (BA1046, Boster) at 4 °C overnight. The protein A/G beads (B23202, Selleck) were washed with IP wash buffer for three times. Antibody-conjugated lysates were incubated with 30 μl protein A/G beads for 2∼4 h. Subsequently, the supernatant was discarded, and the beads were washed three times. Finally, the beads with binding complexes were boiled and subjected to SDS-PAGE for Western blotting. The antibodies used in this study were TLR9 (13674S, CST), EBNA1 (BM1083, OriGene), GAPDH (60004-1-Ig, Proteintech), METTL3 (15073-1-AP, Proteintech), m^6^A (202003, Synaptic Systems), SALL1 (AWA56088, Abiowell),PRKN(AWA10174, Abiowell), MID1 (AWA40807, Abiowell), Myc-tag(60003-2-Ig, Proteintech), TLR3 (AWA50624, Abiowell), TLR7 (AWA58104, Abiowell), TLR8 (AWA58112, Abiowell), ALKBH5 (ab69325, Abcam), FTO (27226-1-AP, Proteintech), GFP (YM3124, Immunoway), α-Tubulin (66031-1-Ig, Proteintech), YTHDF1 (17479-1-AP, Proteintech), YTHDF2 (24744-1-AP, Proteintech), YTHDF3 (25537-1-AP, Proteintech), YTHDC1 (14392-1-AP, Proteintech), TBK1 (38066, CST), p-TBK1 (5483, CST), MYD88 (4283, CST), p-P65 (S536) (3033S, CST), p65 (8242, CST), IRF3 (11904, CST), Histone H3 (A2348, Abclonal), ubiquitin (20326, CST), Goat anti-Rabbit IgG H&L (HRP) (511203, ZEN-Bioscience), Goat anti-Mouse IgG H&L (HRP) (511103, ZEN-Bioscience). The blots quantitatively analyzed by Image J (https://imagej.nih.gov/ij/download.html).

### Reverse transcription-quantitative PCR

Total RNAs from cells were isolated using TRIzol reagent (Invitrogen). Subsequently, 2 μg RNA was reverse transcribed into synthesize complementary DNA in a 20 μl reaction mixture using RevertAid First Strand complementary DNA Synthesis Kit (K1622, Thermo Fisher Scientific). Real-time reverse-transcription PCR was carried out by SYBR premix Ex TaqII Kit (Takara). *Actin* was performed in parallel as a control. The mRNA expression of each gene was quantified by measuring cycle threshold values. The relative expressions were calculated using the 2^−ΔΔCt^ method. The primers for RT-qPCR are shown in Table S2.

### EBV genome copy numbers assay

Total DNA was isolated using the Universal genomic DNA kit（Takara）following the manufacturer's instructions and was primarily quantified with NanoDrop (Thermo Fisher Scientific). One pair of primers (genomic DNA [gDNA]-1) was used to detect the cellular genome. Another pair of primers (EBV-W) was used to detect the viral genome by qPCR analysis. The primer sequences are listed: EBV-W-F: CAGACGAGTCCGTAGAAGGGT; EBV-W-R: TAGGGAACTGAGGAGGGCAT; gDNA-1- F:CCTTTTGTAGGAGGGACTTAGAG; gDNA-1-R: GTATTCACCACCCCACTATGC.

### Dot blot

Cells were collected to extract total RNA or ployA+ RNA. Total RNA was denatured at 65 °C for 10 min and placed on ice. Concentrations of 100 ng, 200 ng, or 500 ng of RNA were spotted on Hybond-N+ membrane and crosslinked for 10 min on a UV crosslinker. The membrane was blocked with 5% skimmed milk for 1 h. Primary m^6^A antibody (1:1000) was incubated overnight at 4 °C. The membrane was washed 3 times with TBST for 10 min each time, and the secondary antibody was incubated at room temperature for 1 h. The membrane was washed three times with TBST for 10 min each time, and chemiluminescence was performed. Finally, the membrane was incubated in 0.02% methylene blue solution for 5 min, and then the membrane was taken out and photographed. The whole process must be RNase-free.

### Flow cytometry

Cells were collected and washed 2 to 3 times. The cell surface mixed antibody was divided equally into each sample staining with the Zombie aqua fixable viability kit (423101, BioLegend), and continued simultaneously for 30 min at room temperature. After the cell surface staining was finished, the intracellular maker staining was performed. Cell fixation (420801, BioLegend) and staining permeabilization (421002, BioLegend) were required prior to intracellular staining. Flow cytometry determined the expression of distinct surface and intracellular molecules. The following antibodies were used: APC/Cyanine7 anti-human CD3 (300317, BioLegend), PerCP/Cyanine 5.5 anti-human CD4 (317427, BioLegend), PE/cyanine7 anti-human CD8a (300913, BioLegend), Brilliant Violet 421 anti-human IFN-γ (502531, BioLegend), and PE/DazzleTM594 anti-human CD107a (LAMP-1) (328645, BioLegend) and APC anti-human TNF-α (502913, BioLegend), APC anti-human CD19 (302211, BioLegend). The apoptosis of BJAB cells was assayed using the FITC Annexin V Apoptosis Detection Kit with PI (640914, BioLegend). The data were analyzedusing FlowJo 10.8 software (https://www.flowjo.com/).

### PBMCs isolation and B-cell proliferation

PBMCs were centrifuged from peripheral blood of healthy donors in a gradient by Ficoll (LTS1077-1, TBD) at room temperature. Subsequently, 2.5 × 10^5^ PBMCs per well were stained with CFSE dyes (423801, BioLegend) and cultured with F(ab’)2-Goat anti-human IgG, IgM (10 mM,16-5099-85, Invitrogen), Human sCD40 Ligand (1 mM, 310-02, PeproTech), and Human IL-4(10 nM, 200-04, PeproTech), co-stimulated with DMSO, STM2457(10 μM), or ODN-2006(5 μM) for 6 days in U-bottom 96-well culture plates. CD19+ B cells stained CFSE dyes were gated by flow cytometry analysis. Additionally, B cells were negatively selected using MojoSort human Pan B cell Isolation Kits (#480081, BioLegend) and analyzed by APC anti-human CD19 (302211, BioLegend) by flow cytometry.

### BJAB cells-T cells Coculture assay

Primary T cells were cultured with IL-2 (50 ng/ml) and ImmunoCult Human CD3/CD28/CD2 T Cell Activator (25 μl/ml, 10970, STEMCELL Technologies) from PBMCs. T cells were expanded according to the manufacturer's protocol (58, 81). In U-bottom 96 wells plate(3799, Corning), 1.2 × 10^6^ T cells were cocultured with 1.5 × 10^5^ BJAB cells (8:1) under different conditions. Total cells were treated with cell activation cocktail (with Brefeldin A) (423304, BioLegend) for 6 h before collected. After 72 h, cell cultures were stained for surface markers with CD3, CD4, and CD8a in PBS (with 2.5% FBS).

### Polysome profiling

Cells were treated with CHX (100 μg/ml) for 10 min in culture medium at 37 °C. Cells were then harvested and lysed on ice with hypotonic buffer and lysis buffer (1:1) for 20 min. Lysates are collected and loaded onto a 15/50% (w/v) sucrose gradient, then, they were centrifuged at 4 × 10^4^ rpm for 2 h at 4 °C. The samples were fractionated and analyzed using Biocomp 152 Instruments (Chongqing International Institute for Immunology). RNA was purified from each fraction and analyzed by RT-qPCR.

### ELISA analysis

Primary B or BJAB cells were treated with DMSO, STM2457 (10 μM), or CpG-ODN2006 (5 μM) with 50 MOI EBV infection for appointed time. ELISA was performed as described previously (82). ELISA kits were obtained from Dakewe: Human IFN-α Precoated ELISA Kit (Cat#:1110012), Human IP-10 Precoated ELISA Kit (Cat#:1117452), Human IgA Precoated ELISA Kit (Cat#:1128172), Human IgM Precoated ELISA Kit (Cat#:1128182), Human IgG Precoated ELISA Kit(Cat#:1128162), Human IL-6 Precoated ELISA Kit(Cat#:1110602), Human IL-8 Precoated ELISA Kit (Cat#:1110802).

### Ubiquitination assays

For *in vitro* ubiquitination assays, GFP-NC and GFP-EBNA1, Flag-METTL3 and plasmids containing various polyubiquitin chains conjugated to the lysine 6 site (K6), K11 site, K27 site, K29 site, K33 site, K48 site, and K63 site, were cotransfected for 48 h. Cells were treated with MG132 (25 μM) for 4 h before harvest. Lysates were immunoprecipitated with anti-Flag beads and the ubiquitinated METTL3 was detected using IP assay. For IP, lysates were incubated with anti-Flag antibody at 4 °C overnight. The protein A/G beads were washed with IP wash buffer for 3 times. Antibody-conjugated lysates were incubated with 30 μl protein A/G beads for 2∼4 h. Subsequently, the supernatant was discarded and the beads were washed three times. Finally, the beads with binding complexes were boiled and subjected to SDS-PAGE for Western blotting.

### Statistical analysis

Statistical analysis was determined by independent *t* test or ANOVA using SPSS17.0 and GraphPad Prism.9.0 (https://www.graphpad.com/). Significance parameters were set at *p* < 0.05.

## Data availability

MeRIP-seq data was analyzed in the manuscript, which has been saved to the Gene Expression Omnibus database under the accession number GSE133936.

## Supporting information

This article contains supporting information.

## Conflict of interest

The authors declare that they have no conflicts of interest with the contents of this article.
