# Supplementary material for: Epstein-Barr virus suppresses N6-methyladenosine modification of TLR9 to promote immune evasion
Source: J Biol Chem. 2024 Mar 25;300(5):107226. doi: 10.1016/j.jbc.2024.107226 (PMC11061751; doi:10.1016/j.jbc.2024.107226)
Supplement: Supplemental Tables S1 and S2 [file mmc1.docx]

**Supplementary Table S1. The sequence for siRNA and ShRNA(5’to3’)**

| Name | Sequence (5’ to 3’) |
| --- | --- |
| siNC | Sense:5’-UUCUCCGAACGUGUCACGUTT-3’ |
|  | Antisense: 5’-ACGUGACACGUUCGGAGAATT-3’ |
| siMETTL3 | Sense:5’-GCAAGUAUGUUCACUAUGATT-3’ |
|  | Antisense:5’- UCAUAGUGAACAUACUUGCAG-3’ |
| siFTO | Sense:5’-GGUGCUCCGUGAAGUUAAATT-3’ |
|  | Antisense:5’-UUUAACUUCACGGAGCACCTT-3’ |
| siYTHDF1 | Sense:5’-GGAUACAGUUCAUGACAAUTT-3’ |
|  | Antisense:5’-AUUGUCAUGAACUGUAUCCTT-3’ |
| siYTHDF2 | Sense:5’-CUGCUUAUCGUUCCAUGAATT-3’ |
|  | Antisense:5’-UUCAUGGAACGAUAAGCAGTT-3’ |
| siYTHDF3 | Sense:5’-CAAUUCAAGGGACACUCAATT-3’ |
|  | Antisense:5’-UUGAGUGUCCCUUGAAUUGTT-3’ |
| siRNF113A | 5’-GACCCACAATCCAATGATA-3’ |
| siTRIM21 | 5’-GCAGAGCAUACCUGGAAAUTT-3’ |
| siSALL1-1 | 5’-GGAAGAGCTTTCACGACTA-3’ |
| siSALL1-2 | 5’-CGAACAGATTCGTCACCAA-3’ |
| siPRKN-1 | 5’-AGTGCAGTGCCGTATTTGA-3’ |
| siPRKN-2 | 5’-TGACCAGTTGCGTGTGATT-3’ |
| siMID1-1 | 5’ -ACCGCATCCTAGTATCACA-3’ |
| siMID1-2 | 5’ -GCAACGTCACCCTACAGAA-3’ |
| siHUWE1 | 5’-GGGAGUGGUAUAUGAUCAUTT-3’ |
| siHERC2 | 5’ -GTGGAATGAAATGGTTAAA-3’ |
| siKDM1B-1 | 5’ -GGGTCTTCAGGATGCCTATTT-3’ |
| siKDM1B-2 | 5’ -TTAACAACCCAGTAGCATTAA-3’ |
| siSYVN1-1 | 5’-CAGGCUUCAUCAAGGUUCUTT-3’ |
| siSYVN1-2 | 5’-AGAACCUUGAUGAAGCCUGTT-3’ |
| siBARD1-1 | 5’ -GTACTAACATTCTGAGAGA-3’ |
| siBARD1-2 | 5’ -GGATGCTACTTCTATTTGT-3’ |
| siBMI1-1 | 5’-CACAACCATAATAGAATGT-3’ |
| siBMI1-2 | 5’-TGCTTTGTGGAGGGTACTT-3’ |
| siYTHDC1 | 5’- CAAGGAGTGTTATCTTAAT-3’ |
| shMETTL3 | 5’-GGGCCCAAGTGCAAGAATTCT-3’ |
| shYTHDF1 | 5’-GGAUACAGUUCAUGACAAUTT-3’ |
| shYTHDF2-2 | 5’- GGCTGGTTCTGGATCTACTCCTTCA-3’ |
| shYTHDF3-1 | 5’-CAATTCAAGGGACACTCAA-3’ |

**Supplementary Table S2. Primers for qPCR**

| Primer name | Sequence (5’ to 3’) |
| --- | --- |
| *TLR9* | F: CTGCCACATGACCATCGAG |
|  | R: GGACAGGGATATGAGGGATTTGG |
| *Actin* | F: GAGCTACGAGCTGCCTGACG |
|  | R: GTAGTTTCGTGGATGCCACAG |
| *METTL3* | F: TTGTCTCCAACCTTCCGTAGT |
|  | R: CCAGATCAGAGAGGTGGTGTAG |
| *YTHDF1* | F: ACCTGTCCAGCTATTACCCG |
|  | R: TGGTGAGGTATGGAATCGGAG |
| *EBNA1* | F: CACCATTGAGTCGTCTCCCC |
|  | R: GTAGGAGCGGGCTTTGTCAT |
| *EBNA2* | F: TCTGCCACCTGCAACACTAA |
|  | R: GTCTGGCACATGCAAGACA |
| *GAPDH* | F: GGAGCGAGATCCCTCCAAAAT |
|  | R: GGCTGTTGTCATACTTCTCATGG |
| *TLR1* | F: CCACGTTCCTAAAGACCTATCCC |
|  | R: CCAAGTGCTTGAGGTTCACAG |
| *TLR2* | F: ATCCTCCAATCAGGCTTCTCT |
|  | R: GGACAGGTCAAGGCTTTTTACA |
| *TLR3* | F: TTGCCTTGTATCTACTTTTGGGG |
|  | R: TCAACACTGTTATGTTTGTGGGT |
| *TLR4* | F: AGACCTGTCCCTGAACCCTAT |
|  | R: CGATGGACTTCTAAACCAGCCA |
| *TLR5* | F: GCCGGTCCTGTGTTTGGAAT |
|  | R: GGTGAGGTTGCAGAAACGATAAA |
| *TLR6* | F: TTCTCCGACGGAAATGAATTTGC |
|  | R: CAGCGGTAGGTCTTTTGGAAC |
| *TLR7* | F: TCCTTGGGGCTAGATGGTTTC |
|  | R: TCCACGATCACATGGTTCTTTG |
| *TLR8* | F: ATGTTCCTTCAGTCGTCAATGC |
|  | R: TTGCTGCACTCTGCAATAACT |
| *TLR10* | F: AGGTTTGAGTGGGGCAAAAAT |
|  | R: CCATCACGCAAAAGAACCCAG |
| *BARD1* | F: CTGCTCGCGTTGTACTAACAT |
|  | R: TCCAATGCAGTCACTTACACAAT |
| *MID1* | F: CTGACCTGCCCTATTTGTCTG |
|  | R: GCACAGTGTGATACTAGGATGC |
| *PRKN* | F: GTGTTTGTCAGGTTCAACTCCA |
|  | R: GAAAATCACACGCAACTGGTC |
| *SALL1* | F: CCCCGGTTGCTAACAAAAGC |
|  | R: GAGGTTGTGATCGCTGAGGTA |
| *SYVN1* | F: GCTCACGCCTACTACCTCAAA |
|  | R: GCCAGACAAGTCTCTGTGACG |
|  |  |
|  |  |
| *RNF113A* | F: CTGTGGTTCGACCGGAAAAGA |
|  | R: GCTGCTCAAGTCGCCGTAA |
| *TRIM28* | F: TTTCATGCGTGATAGTGGCAG |
|  | R: GCCTCTACACAGGTCTCACAC |
| *MYCBP2* | F: GGGGACGGATTCTACCCAG |
|  | R: ATTGAGCGCAGCGGTATAAAT |
| *UBR5* | F: GTCCATCCATTTCGTGGTTCA |
|  | R: CCAATTCCAATCTGTCTGGCTG |
| *HERC2* | F: TCGCCTCGACTCCAAATGG |
|  | R: TCTTTGTTCCACTTGGTTCGAC |
| *TRIM41* | F: CTGCCGAGTTTGTGTAACCCA |
|  | R: CTCCTCCATGTCACCCTCGTA |
| *HUWE1* | F: TTGGACCGCTTCGATGGAATA |
|  | R: TGAAGTTCAACACAGCCAAGAG |
| *TRIM21* | F: TCAGCAGCACGCTTGACAAT |
|  | R: GGCCACACTCGATGCTCAC |
| *KDM1B* | F: GACTAGGTTCGGTTTTGCCATT |
|  | R: CTCTCCTGTGGGGAACATTTC |
