# Supplementary material for: Epstein-Barr virus suppresses N6-methyladenosine modification of TLR9 to promote immune evasion
Source: J Biol Chem. 2024 Mar 25;300(5):107226. doi: 10.1016/j.jbc.2024.107226 (PMC11061751; doi:10.1016/j.jbc.2024.107226)
Supplement: Supplemental Figures S1–S6 [file mmc3.docx]

Epstein-Barr virus suppresses N^6^-Methyladenosine modification of TLR9

to promote immune evasion


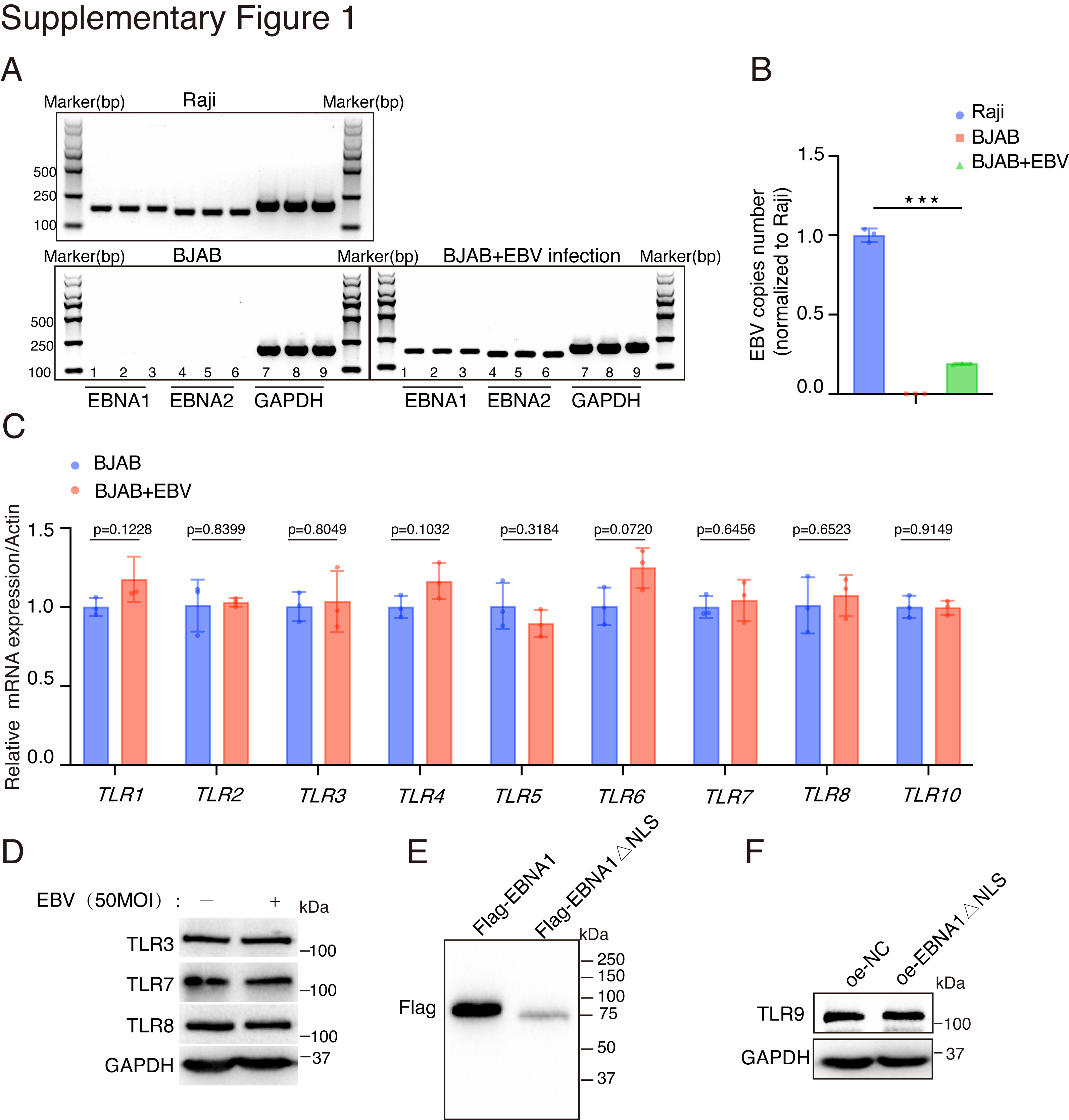


Supplementary Fig.S1. (A-D) BJAB cells were infected with EBV (50MOI) for 48h. (A) The expression levels of EBNA1, EBNA2 and GAPDH were detected by RT-qPCR. 2% agarose gel electrophoresis was performed. RT-qPCR was tested on 3 biological replicates. Raji cell is an EBV positive cell line, and BJAB cell is an EBV negative cell line. (B) EBV copies number were assayed by qPCR analysis. Raji cells were used as a positive control. (C) mRNA expression levels of TLR genes were examined by RT-qPCR. (D) Protein expression levels of TLR family were measured by Western blotting. (E, F) HEK293 cells were transfected with plasmids containing Flag-NC, Flag-EBNA1 or Flag-EBNA1ΔNLS for 48 h. Experiments were independently repeated three times, and results are presented as mean ± SD. ****p <* 0.001.


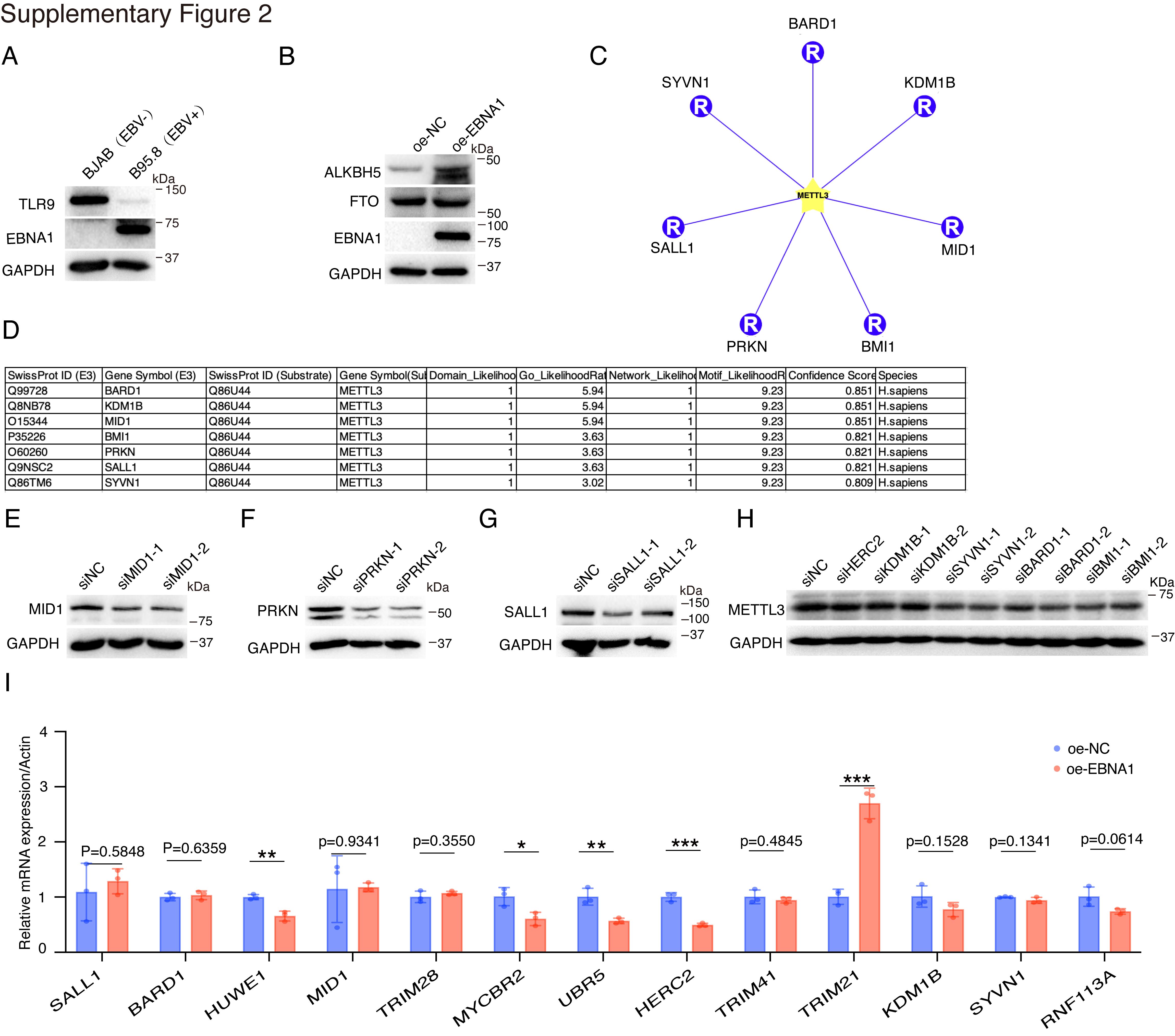


Supplementary Fig.S2.(A) BJAB(EBV-negative cell line) and B95.8 (EBV-positive cell line）cells were collected. The protein expression of TLR9 and EBNA1 were tested by Western blotting. (B) HEK293 cells transfected with plasmids encoding Flag-NC or Flag-EBNA1 for 48 h. The indicated proteins were assayed. (C, D) The predicted interactions between the ubiquitin ligases and the substrate (METTL3) in eukaryotic species by means of UbiBrowser 2.0. (E-H) HONE1 cells were transfected with indicated siRNAs for 48 h. (I) HONE-1 cells were transfected with plasmids encoding Flag-NC or Flag-EBNA1 for 48 h. The results are presented as mean ± SD. **p <* 0.05, ***p <* 0.01, ****p <* 0.001.


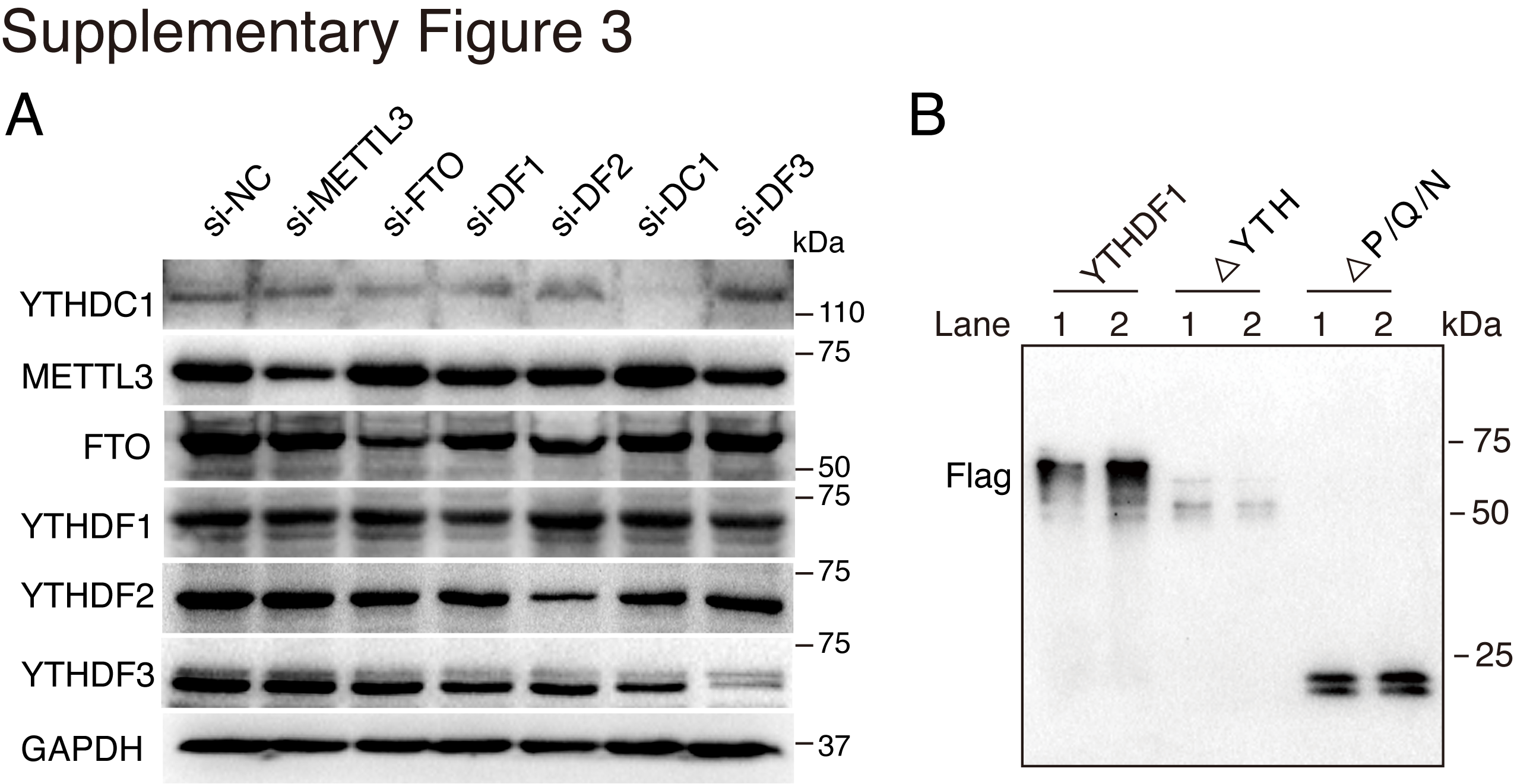


Supplementary Fig.S3. (A) HEK293 cells were transfected with indicated siRNAs for 48 h. The proteins expression levels were tested by Western blotting. (B) HEK293 cells were transfected with Flag-YTHDF1, Flag-△YTH and Flag-△P/Q/N plasmids for 48 h. Cell lysate was assayed by Western blotting with anti-Flag antibody. These data are shown as the mean ± SD. Lane 1, 2: two repeats.


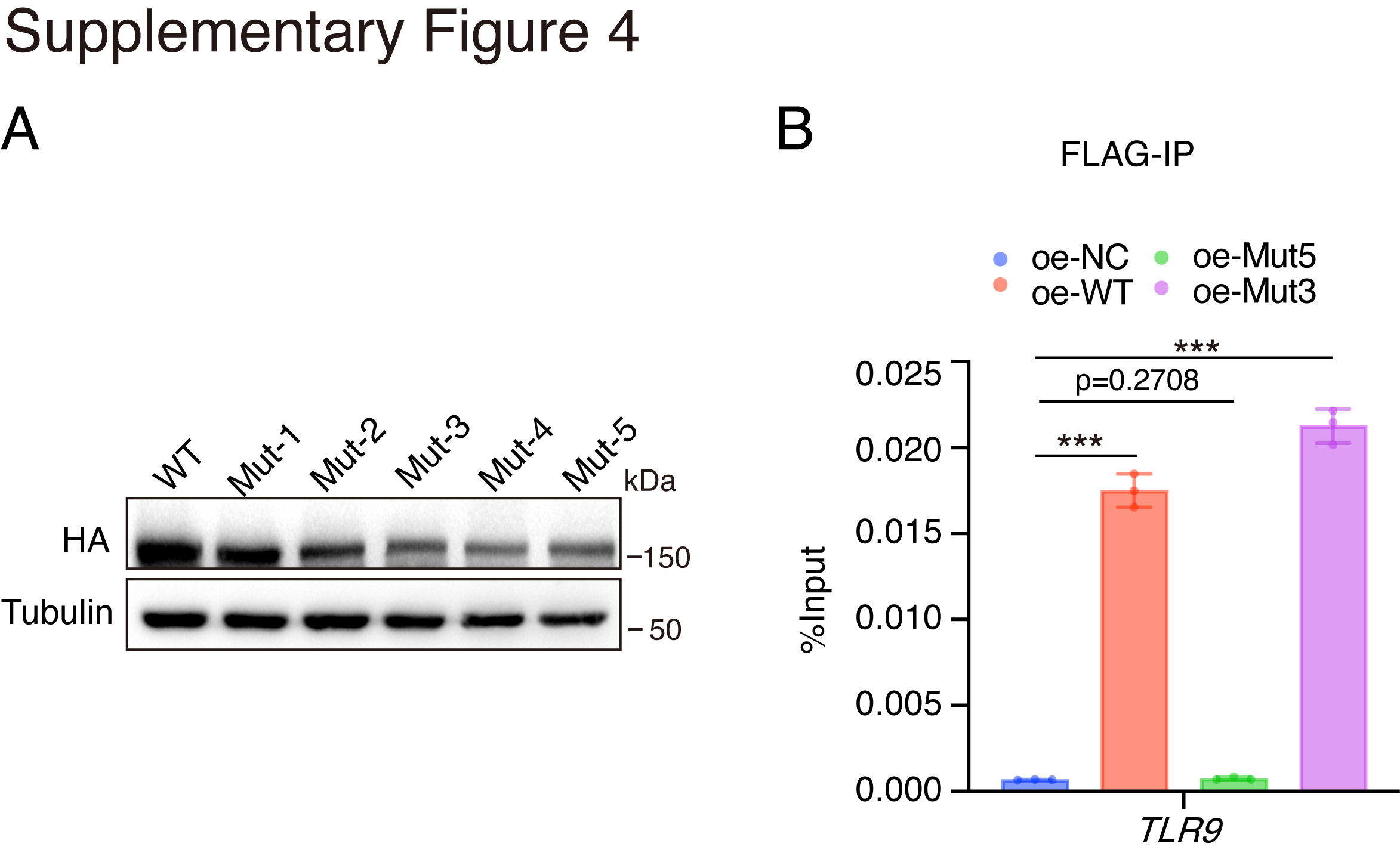


Supplementary Fig.S4. (A) HA-TLR9 plasmids (WT or mutants) were transfected into HEK293 cells for 48 h. (see Fig. 5E for TLR9 plasmids information) (B) RNA immunoprecipitation analysis of HEK293 cells transfected for 48 h with plasmids encoding FLAG-YTHDF1 and HA-TLR9 WT or HA-TLR9 m^6^A mutants to test TLR9 mRNA-YTHDF1 binding. These data are shown as the mean ± SD. ****p<* 0.001.


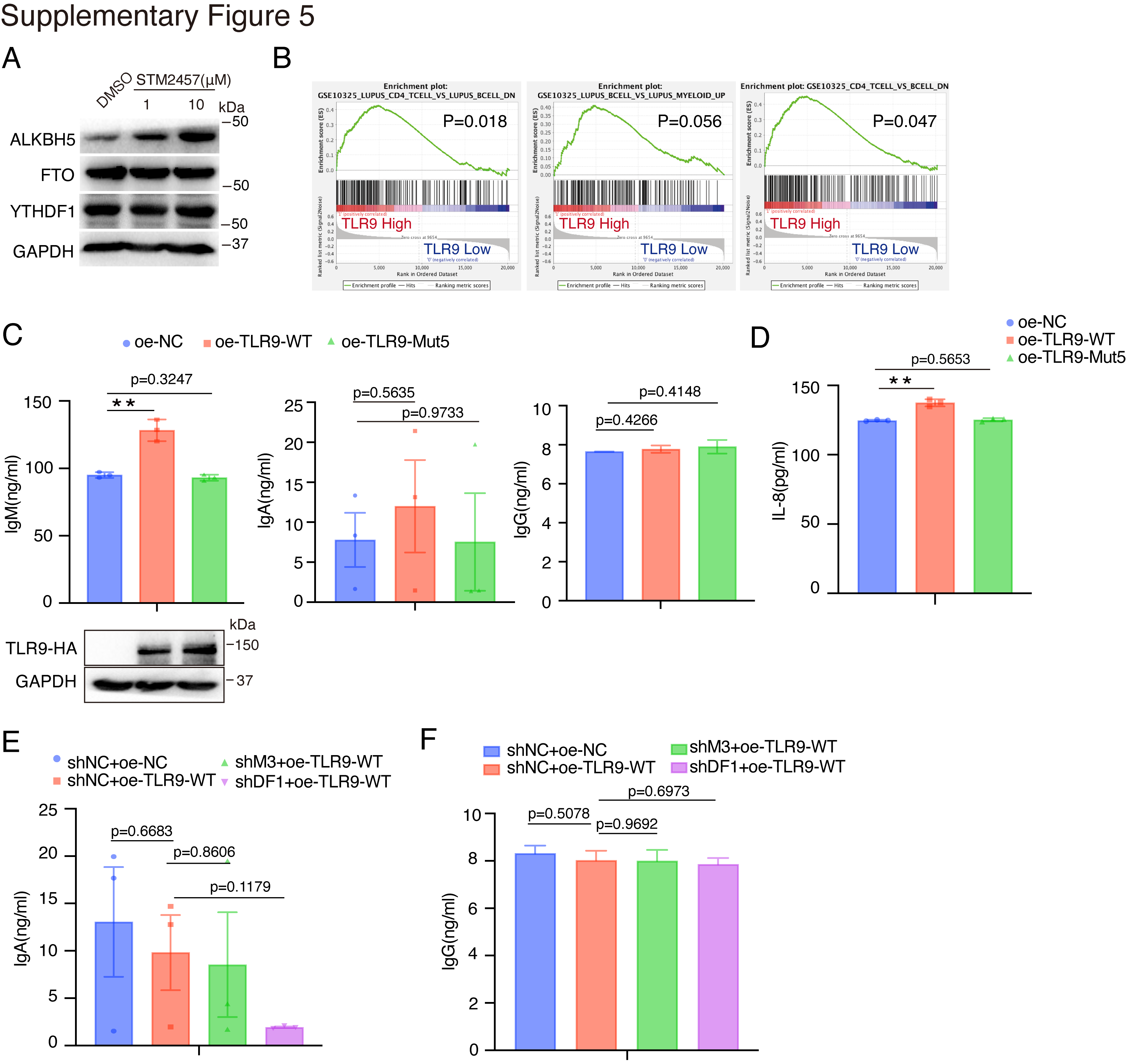


Supplementary Fig.S5. (A) BJAB cells were treated with STM2457 or DMSO (as control) for 96 h. Western blotting assay was performed. (B) Gene sets difference between the TLR9 high and low whole blood or mononuclear cell samples from patients with systemic lupus erythematosus revealed by GSEA. The top gene sets enriched in TLR9 high group comparing to low group are listed. (C, D) BJAB cells were transfected with HA-TLR9WT or HA-TLR9 Mut 5 plasmids for 48 h, and the supernatants of cells were analyzed for ELSIA. (E, F) BJAB cells were transfected with indicated shRNAs and expression plasmids for 48 h, and the supernatants of cells were analyzed for ELSIA. These data are shown as the mean ± SD. ***p <* 0.01.


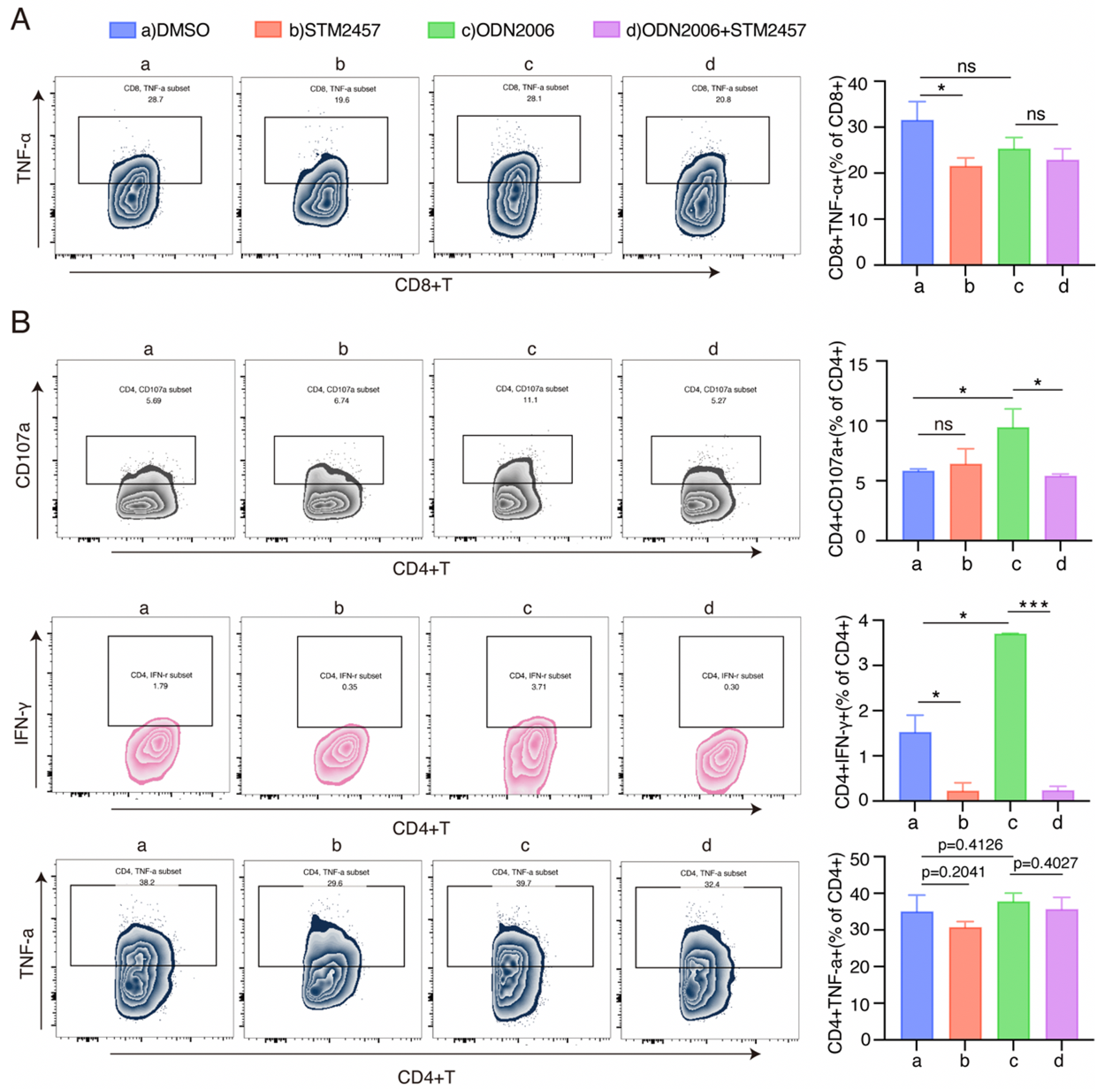


Supplementary Fig.S6. BJAB cells were co-cultured with primary T-cell (T cells: BJAB cells =8:1) in supernatants containing IL-2 (50ng/mL) under different treatment conditions as indicated. Total cells were treated with cell activation cocktail (with Brefeldin A) for 6 h before collected, then, detected by flow cytometry. Representative histograms of CD8^+^ TNF-α^+^ T cells (A), CD4^+^ CD107a^+^ T cells, CD4^+^IFN-γ^+^ T cells and CD4^+^TNF-α^+^ T cells (B) were shown. **p <* 0.05, ****p <* 0.001, ns, not significant.
